# Evaluation of the Phenolic Profile and Antioxidant Capacity of Landraces and Modern Tunisian Durum Wheat Genotypes Under Organic Farming System

**DOI:** 10.1002/fsn3.72356

**Published:** 2026-09-23

**Authors:** Anis Boukrain, Chirine Brinsi, Mondher Mejri

**Affiliations:** ^1^ Higher Institute of Biotechnology of Beja, Laboratory of Functional Physiology and Valorization of Bio‐Resources (LR23ES08) University of Jendouba Beja Tunisia

**Keywords:** antioxidant, durum wheat, FRAP, organic farming, phenolic acids, polyphenols

## Abstract

The consumption of whole wheat has increased due to its nutritional benefits, particularly its richness in phenolic compounds. This study evaluated the influence of the farming systems (organic and conventional) and the diversity of thirteen Tunisian durum wheat varieties (landraces and modern genotypes) on the phenolic profile and antioxidant capacity. UHPLC–MS^n^ analysis allowed the identification of eight phenolic acids, mainly in bound form, while p‐coumaric acid was the only compound detected in its free form. The results showed a highly significant effect of both the farming system and the variety on the bound and total fractions of all identified acids, on the total phenolic content and on the total antioxidant capacity, highlighting a combined genetic and environmental influence on their accumulation. On average, antioxidant capacity measured using the FRAP assay reached 2.70 mmol Fe/100 g under conventional farming and 2.90 mmol Fe/100 g under organic farming, indicating a slight increase in the organic system. The varieties Jenah Khotifa and Chili exhibited the highest values, reaching 3.65 mmol Fe/100 g and 3.38 mmol Fe/100 g, respectively, in the organic system, and 3.04 mmol Fe/100 g for Chili in the conventional system. Genotype and farming system influence phenolic profile. Organic farming enhanced the accumulation of bioactive compounds and increased antioxidant capacity, particularly in landraces Chili, Biskri, and Jenah Khotifa, compared to modern varieties. These findings highlight the value of traditional genotypes and organic farming in improving the functional quality of Tunisian durum wheat, while preserving local cereal biodiversity and enhancing nutritional quality.

## Graphical abstract

This study evaluated the effects of organic and conventional farming systems on the phenolic profile and antioxidant capacity of 13 Tunisian durum wheat varieties, including traditional landraces and modern genotypes. Phenolic compounds were characterized by UHPLC–MS, while antioxidant capacity was assessed using the FRAP assay. Organic farming enhanced phenolic compound accumulation and antioxidant activity, with particularly pronounced responses observed in traditional landraces. These findings highlight the potential of Tunisian durum wheat genetic resources for functional wheat development, nutritional breeding, and sustainable agriculture.

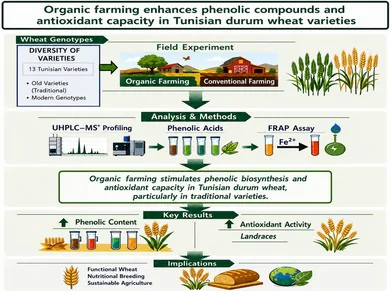

## 1 Introduction

The growing consumption of whole grains is part of a global trend driven by increasing consumer awareness of healthier diets, and interest in secondary metabolites of cereals, such as polyphenols, continues to rise. These phytometabolic compounds play a key role in the health benefits associated with whole grain consumption (Khan et al. 2022), particularly through their antioxidant activity and their involvement in the prevention of chronic diseases including cardiovascular disorders, Type II diabetes, obesity, and certain cancers (Khan et al. 2024). Dietary phenolic compounds have recently attracted considerable attention from nutritionists, food technologists, and consumers (Tsao 2010), and growing scientific evidence links this class of phytochemicals with a wide range of biological activities (Del Rio et al. 2013; Rodriguez‐Mateos et al. 2014). Phenolic acids represent the most common form of phenolic compounds in whole grains and constitute one of the major and most complex groups of phytochemicals in cereal kernels (Li et al. 2008). Wheat phenolics occur in free, bound, and soluble conjugated forms (Ghasemzadeh and Ghasemzadeh 2011), as well as in oligomeric forms as described by Braune et al. (2009). However, phytochemicals have received limited attention from plant breeders. Traditionally, breeding programs have targeted technological quality traits, productivity, and resistance to biotic and abiotic stresses (Leoncini et al. 2012), whereas improvements in nutritional quality have been considered a lower priority. The development of wheat varieties with enhanced health‐promoting attributes is regarded as a complex objective for many countries (Hakeem et al. 2025). In durum wheat ( *Triticum durum* Desf.), the primary cereal cultivated in the Mediterranean region, breeding efforts have mainly focused on key agronomic and technological traits such as grain yield, protein content, and gluten quality, while micronutrients and secondary metabolites have been comparatively overlooked (Cabas‐Lühmann et al. 2023). Yet, the genetic diversity preserved in landraces and ancient varieties represents a valuable resource for improving the nutritional and functional quality of new cultivars (Boukid et al. 2019). Despite extensive research on the health benefits of whole grains, limited information is available regarding the impact of modern breeding strategies on phenolic content (Li et al. 2008; Nigro et al. 2017; Pasqualone et al. 2014). Such investigations could provide essential insights for identifying the most suitable varieties to produce final products with enhanced nutritional properties and reduced risk of undesirable traits (Nigro et al. 2017; Pasqualone et al. 2014). Since selection directly affects major grain components such as proteins and starch, the quality and quantity of polyphenols may also be influenced. Therefore, understanding the genetic heritability of phenolic compounds could yield valuable results (De Vita et al. 2007; Dinelli et al. 2011).

In this context, it is crucial to determine how total phenolic content, phenolic profile, and total antioxidant capacity vary according to the farming system and the genotype. Organic farming, by modifying plant‐environment interactions and inducing controlled abiotic stress, has the potential to promote the accumulation of beneficial phenolic compounds (Carucci et al. 2023). The present study investigated 13 Tunisian durum wheat varieties, including a historical panel composed of landraces and modern genotypes, grown under two farming systems: conventional and organic. The objective was to assess the combined influence of the farming system and genotype on total phenolic content, phenolic profile and total antioxidant capacity, with the ultimate aim of providing new insights and relevant data to support the selection of varieties that are productive, nutritionally enhanced and well adapted to sustainable farming systems.

## 2 Material and Methods

### 2.1 Plant Material

Thirteen durum wheat ( *Triticum durum* Desf.) varieties, representative of Tunisian populations cultivated throughout the 20th century, were selected for this study (Table 1). These varieties exhibit substantial genetic diversity and were classified into two groups: local varieties (*n* = 4) and modern varieties (*n* = 9). The selection aimed to evaluate their performance under organic farming systems. Specific characteristics of each genotype are provided in Table 1.

**TABLE 1:** Origin, year of registration, and main characteristics of the thirteen durum wheat genotypes studied (Boukrain, Brinsi, and Mejri 2026; Gharbi and El Felah 2013; Ammar et al. 2011; Deghaïs et al. 2007).

Genotypes	Origin	Registration date	Main characteristics
Karim	INRAT/CIMMYT	1980	Fairly resistant to septoria and brown rust
Razzak	INRAT‐Tunisia	1987	Very productive cultivar (25% more than Karim), resistant to powdery mildew and fairly resistant to septoria and brown rust
Khiar	INRAT/CIMMYT	1992	Cultivars that are productive and relatively susceptible to brown rust and septoria diseases
Maali	INRAT‐Tunisia	2007	Resistant to powdery mildew and fairly resistant to septoria and brown rust
Salim	INRAT‐Tunisia	2009	High yield, resistance to septoria, yellow rust, and brown rust
INRAT100	CIMMYT/Tunisia	2017	Cultivars that are productive, resistance to septoria, yellow rust, and brown rust, and drought tolerance
Dhahbi	INRAT‐Tunisia	2018	Productive, resistant cultivar to powdery mildew and yellow rust and very high semolina quality
Monastir	SOSEM	2012	Cultivars that are productive, resistant to septoria and brown rust, and tolerant to drought
Saragolla	Italy	2004	High yield, resistance to septoria and rust
Chili	French (old variety/landrace)	1932	Medium yield, resistance to septoria and yellow rust
Biskri	Algeria (old variety/landrace)	1909	Medium yield, drought tolerance and very high semolina quality
Mahmoudi	Tunisia (old variety/landrace)	1893	Medium yield, drought tolerance and very high semolina quality
Jenah Khotifa	Tunisia (old variety/landrace)	1915	Medium yield, resistance to septoria and yellow rust

Abbreviations: CIMMYT, International Maize and Wheat Improvement Centre; ICARDA, International Center for Agricultural Research in the Dry Areas; INRAT, National Institute of Agricultural Research of Tunisia; SOSEM, Selected Seeds Company (Tunisia).

### 2.2 Field Experimental Conditions and Crop Management

The comparative evaluation of the varieties was conducted under field conditions during the 2023–2024 growing season at the Gnadil experimental station, located in Beja, Tunisia (36°44′05″ N, 9°13′35″ E). The site is characterized by a Mediterranean climate corresponding to the subhumid bioclimatic zone, with cold winters and very hot, dry summers. During the cropping season, cumulative precipitation reached 528 mm, with an average minimum temperature of 8.5°C and an average maximum temperature of 26.9°C. The experimental soil was characterized as a clay‐loam Vertisol with a pH of 7.2. The trial was established using a split‐plot design with three replications. The main factor corresponded to the farming system (organic vs. conventional), while the varieties, randomized within each treatment, constituted the subplot factor. Each replication comprised two subplots, one managed under conventional farming and the other under organic farming. Elementary plots measured 3 m × 1 m and included six rows spaced 20 cm apart, resulting in a total trial area of 234 m^2^. Before the establishment of the experiment, the field was maintained under fallow conditions for three consecutive years to minimize the influence of previous crops and ensure homogeneous initial conditions. This period also contributed to maintaining the organic status of the experimental field. Soil preparation consisted of moderate plowing using an offset disc harrow, followed by incorporation of crop residues and seedbed preparation using a cultivator and a harrow.

Sowing was carried out during the second half of November at a density of 600 seeds per elementary plot, corresponding to an average seeding rate of 120 kg ha^−1^. Weed management initially involved stubble cultivation followed by false sowing. Subsequent weed control strategies were adapted according to the farming system: in organic plots, weed control was performed through mechanical interventions using a rotavator combined with manual weeding using a rake and hoe, whereas in conventional plots, chemical weed control was performed by herbicide application (Granstar, 20 g ha^−1^) using a backpack sprayer.

Nitrogen fertilization was managed according to the farming system. In conventional plots, nitrogen was supplied as ammonium nitrate (33.5%) at a total rate of 150 kg N ha^−1^, applied in three split doses (30%, 40%, and 30%) after emergence, at the beginning of tillering, and at the second‐node stage. In organic plots, a liquid organic nitrogen fertilizer containing 5.9% organic nitrogen, 22.2% organic carbon, and 36.9% amino acids was applied during the crop cycle. In addition, foliar amino acid fertilization was performed in organic plots at a rate of 2 L ha^−1^, with five successive applications after emergence at 10‐day intervals.

No phytosanitary treatments against fungal diseases or insect pests were applied throughout the entire experimental period.

It is noteworthy that the field had not received any synthetic chemical inputs for at least three years, in accordance with organic farming standards.

### 2.3 Extraction of Free and Bound Phenolic Compounds

Free and bound phenolic compounds from wheat samples were extracted according to the method of González‐Barrio et al. (2010). Briefly, 50 mg of whole wheat flour was mixed with 2 mL of methanol/acidified water (0.1% formic acid) in an 80:20 (v/v) ratio and sonicated for 90 min. The sample was then vortexed for 1 min and centrifuged at 4000 rpm for 10 min. The resulting supernatant was collected and used for the analysis of free phenolic compounds.

The pellet obtained after this initial extraction was hydrolyzed with 1.5 mL of 2 M sodium hydroxide at room temperature for 1 h. Following alkaline hydrolysis, the pH of the mixture was adjusted to 3 by adding 1.35 mL of 3 M citric acid. Bound phenolic compounds were then extracted with 4 mL of ethyl acetate. After centrifugation at 4000 rpm for 10 min, 1 mL of the ethyl acetate supernatant was dried under vacuum using a rotary evaporator, and the pellet was subsequently dissolved in 0.5 mL of methanol/acidified water (0.1% formic acid) (80:20 v/v) for UHPLC‐MSn analysis.

### 2.4 Determination of Total Polyphenol Content

The total phenolic content of free and bound extracts was determined using the Folin–Ciocalteu method as described by Singleton et al. (1999), with modifications according to Mena et al. (2013). Results are expressed as mg of ferulic and gallic acid equivalents (FAE and GAE) per 100 g of whole wheat flour.

### 2.5 UHPLC/MSn Analyses

Phenolic acids in both free and bound extracts were analyzed using an Accela UHPLC 1250 system coupled to a linear ion‐trap mass spectrometer (LTQ XL, Thermo Fisher Scientific Inc., San Jose, CA, USA) equipped with a heated electrospray ionization probe (H‐ESI‐II; Thermo Fisher Scientific Inc.). Chromatographic separation was performed on a BlueOrchid C18 column (50 × 2 mm, 1.8 μm particle size; Knauer, Berlin, Germany). Phenolic acids were detected in negative ionization mode. The mobile phase, delivered at a flow rate of 0.3 mL/min, consisted of acidified water (0.1% formic acid) (solvent A) and a methanol/water mixture (98:2, v/v) (solvent B). After an initial hold of 0.5 min at 7% B in A, the proportion of solvent B was linearly increased to 50% over 6.5 min, followed by 2 min at 80% B and a 4 min return to initial conditions to re‐equilibrate the column. The H‐ESI‐II interface was set to a capillary temperature of 275°C and a source heater temperature of 40°C. The sheath gas (N_2_) and auxiliary gas (N_2_) flow rates were set to 40 and 5 arbitrary units, respectively. During analysis, the source voltage was 4 kV, while the capillary and tube lens voltages were −21 V and −58 V, respectively. Analyses were performed in full MS^2^ mode, monitoring specific ions fragmented by collision‐induced dissociation (CID) using pure helium (99.99%) at 30 units. Diferulic acids at m/z 385 were further characterized by MS^3^ experiments, monitoring the main MS^2^ ion at m/z 341 (CID = 28) which was subjected to additional fragmentation (CID = 35) to confirm the identification of diferulates. Quantification was carried out using calibration curves constructed with commercially available standards of caffeic acid, ferulic acid, p‐coumaric acid, and sinapic acid. Data acquisition and processing were performed using Xcalibur software (Thermo Scientific Inc., Waltham, MA, USA).

### 2.6 Determination of Total Antioxidant Capacity

The total antioxidant capacity of free and bound phenolic extracts was measured using the ferric reducing antioxidant power (FRAP) assay, according to the method described by Adom and Liu (2002).

### 2.7 Statistical Analysis

All statistical analyses were performed using the Statistical Package for the Social Sciences (SPSS), version 27 (IBM Corp., Armonk, NY, USA).

Data are presented as mean ± standard deviation (SD). An analysis of variance (ANOVA) was performed using the General Linear Model (GLM) procedure to evaluate the effects of farming system (two levels: organic vs. conventional), variety (*n* = 13), and their interaction. Following this analysis, significantly different means were compared using Tukey's post hoc test. Principal component analysis (PCA) was used to determine the position index based on multiple variables (total phenolic compounds, identified phenolic acids, and FRAP) in order to explore the clustering patterns among samples. Hierarchical cluster analysis was conducted using the between‐groups linkage method and squared Euclidean distance. Statistical significance was set at *p* < 0.05.

## 3 Results

### 3.1 Free, Bound, and Total Polyphenol Contents of Durum Wheat Varieties

The total polyphenol content varied markedly according to variety and farming system. The ANOVA results showed that the farming system had a highly significant effect (*p* ≤ 0.001) on the free, bound, and total polyphenol fractions of durum wheat varieties, whether expressed as ferulic acid or gallic acid equivalents per 100 g of grain (Table 2 and Appendix S1). Similarly, the variety had a significant influence on the bound and total fractions (*p* ≤ 0.001), while its effect on the free fraction was not significant for ferulic acid (*p* > 0.05) but significant for gallic acid (*p* ≤ 0.001). The farming system interaction was also highly significant (*p* ≤ 0.001) for the bound and total fractions in both acids, whereas it was not significant for the free ferulic acid fraction, indicating a differential genotypic response to farming systems, particularly pronounced for bound compounds.

**TABLE 2:** Free, bound, and total polyphenol contents of durum wheat varieties (mg ferulic acid and mg gallic acid per 100 g of whole wheat grain).

Varieties	Farming system	Free		Bound		Total	
		mg ferulic acid/100 g of grain	mg gallic acid/100 g of grain	mg ferulic acid/100 g of grain	mg gallic acid/100 g of grain	mg ferulic acid/100 g of grain	mg gallic acid/100 g of grain
Karim	CF	10.14 ± 0.23^a^	9.92 ± 0.05^a^	270.44 ± 10.90^a^	240.16 ± 3.75^a^	280.58 ± 11.10^a^	250.08 ± 3.80^a^
	OF	10.02 ± 0.15^b^	9.90 ± 0.02^a^	262.20 ± 7.54^b^	231.38 ± 2.90^b^	273.22 ± 7.69^a^	241.28 ± 2.92^b^
Razzak	CF	9.91 ± 0.10^b^	9.00 ± 0.08^b^	263.92 ± 11.74^b^	220.65 ± 4.67^a^	273.83 ± 11.79^a^	229.65 ± 4.74^a^
	OF	10.13 ± 0.37^a^	9.55 ± 0.03^a^	270.02 ± 6.63^a^	221.47 ± 3.20^a^	280.14 ± 6.98^a^	231.02 ± 3.20^a^
Khiar	CF	9.76 ± 0.05^a^	7.97 ± 0.04^a^	259.74 ± 8.10^a^	174.49 ± 0.62^a^	269.51 ± 8.15^a^	182.46 ± 0.66^a^
	OF	8.63 ± 0.09^b^	8.01 ± 0.02^a^	239.19 ± 3.14^b^	170.61 ± 2.69^b^	247.81 ± 3.23^b^	178.62 ± 2.72^a^
Maali	CF	10.23 ± 0.05^a^	8.54 ± 0.03^a^	292.22 ± 1.80^a^	190.29 ± 2.83^a^	302.44 ± 1.82^a^	198.83 ± 2.87^a^
	OF	9.62 ± 0.10^b^	8.13 ± 0.18^b^	289.11 ± 1.32^a^	179.94 ± 2.33^b^	298.73 ± 1.42^b^	188.07 ± 2.51^b^
Salim	CF	8.62 ± 0.10^a^	8.12 ± 0.02^a^	218.05 ± 5.35^a^	180.32 ± 3.78^a^	226.67 ± 5.44^a^	188.44 ± 3.80^a^
	OF	8.20 ± 0.08^b^	7.57 ± 0.05^b^	219.66 ± 1.54^a^	166.06 ± 2.58^b^	227.86 ± 1.62^a^	173.63 ± 2.63^b^
INRAT 100	CF	10.97 ± 0.05^a^	8.67 ± 0.05^a^	305.63 ± 4.08^a^	199.52 ± 1.99^a^	316.60 ± 4.13^a^	208.20 ± 1.95^a^
	OF	9.87 ± 0.15^b^	7.67 ± 0.05^b^	290.28 ± 1.91^b^	176.23 ± 4.32^b^	300.15 ± 2.06^b^	183.91 ± 4.37^b^
Dhahbi	CF	11.62 ± 0.10^a^	8.94 ± 0.03^a^	339.98 ± 10.53^a^	202.48 ± 5.19^a^	351.60 ± 10.45^a^	211.42 ± 5.22^a^
	OF	10.11 ± 0.06^b^	7.87 ± 0.05^b^	292.81 ± 2.42^b^	180.44 ± 2.16^b^	302.92 ± 2.46^b^	188.31 ± 2.11^b^
Monastir	CF	9.92 ± 0.05^a^	8.47 ± 0.05^a^	270.70 ± 5.84^a^	181.51 ± 4.43^a^	280.62 ± 5.89^a^	189.98 ± 4.49^a^
	OF	9.46 ± 0.44^b^	7.47 ± 0.05^b^	271.51 ± 9.17^a^	178.00 ± 1.23^b^	280.97 ± 9.61^a^	185.47 ± 1.27^a^
Saragolla	CF	10.12 ± 0.05^b^	9.07 ± 0.05^a^	268.26 ± 0.65^b^	209.15 ± 2.06^a^	278.38 ± 0.68^b^	218.23 ± 2.10^a^
	OF	10.27 ± 0.05^a^	8.93 ± 0.01^b^	282.94 ± 1.09^a^	195.27 ± 1.56^b^	293.22 ± 1.13^a^	204.20 ± 1.57^b^
Chili	CF	11.26 ± 0.05^b^	10.14 ± 0.03^a^	306.43 ± 8.25^b^	249.41 ± 3.15^b^	317.69 ± 8.30^b^	259.56 ± 3.18^b^
	OF	11.61 ± 0.09^a^	10.03 ± 0.01^b^	324.66 ± 4.51^a^	288.45 ± 8.35^a^	336.27 ± 4.52^a^	298.48 ± 8.36^a^
Biskri	CF	11.61 ± 0.08^b^	8.77 ± 0.04^b^	293.83 ± 2.39^b^	200.20 ± 2.71^b^	305.44 ± 2.47^b^	208.96 ± 2.74^b^
	OF	12.04 ± 0.02^a^	9.22 ± 0.01^a^	335.42 ± 3.53^a^	262.53 ± 4.11^a^	347.46 ± 3.54^a^	271.75 ± 4.10^a^
Mahmoudi	CF	11.62 ± 0.10^a^	9.15 ± 0.03^b^	297.41 ± 5.27^b^	223.46 ± 4.13^b^	309.03 ± 5.33^b^	232.61 ± 4.15^b^
	OF	11.68 ± 0.15^a^	10.13 ± 0.01^a^	310.10 ± 29.70^a^	285.03 ± 4.79^a^	321.78 ± 29.85^a^	295.16 ± 4.79^a^
Jenah Khotifa	CF	10.92 ± 0.10^a^	8.97 ± 0.05^b^	276.61 ± 2.33^b^	197.51 ± 2.11^b^	287.53 ± 2.43^b^	206.49 ± 2.15^b^
	OF	10.95 ± 0.16^a^	9.07 ± 0.05^a^	302.94 ± 13.27^a^	241.66 ± 0.88^a^	313.88 ± 13.41^a^	250.73 ± 0.83^a^
**Mean**	CF	**10.52 ± 0.88** ^a^	**8.90 ± 0.60** ^a^	**281.79 ± 29.21** ^a^	**205.32 ± 22.36** ^b^	**292.30 ± 29.98** ^b^	**214.22 ± 22.94** ^b^
	OF	**10.20 ± 1.12** ^b^	**8.73 ± 0.96** ^b^	**283.91 ± 32.43** ^a^	**213.62 ± 43.28** ^a^	**294.11 ± 33.46** ^a^	**222.36 ± 44.14** ^a^
*ANOVA*							
Farming system		***	***	***	***	***	***
Variety		NS	***	***	***	***	***
FS × variety		NS	***	***	***	***	***

*Note:* Values are expressed as mean ± standard deviation (*n* = 3). Different letters within the same row indicate statistically significant differences (*p* ≤ 0.05, Tukey's test). The bold values correspond to the mean values calculated across all varieties for each of the two farming systems, namely Conventional Farming (CF) and Organic Farming (OF). The *** indicates a significant effect at the 0.1% probability level.

Abbreviations: CF, conventional farming; OF, organic farming.

The quantification of the total polyphenol content of the free and bound fractions is shown in Table 2. For ferulic acid, values ranged, under organic farming, from 8.20 mg/100 g (Salim) to 12.04 mg/100 g (Biskri), while under conventional farming they ranged from 8.62 mg/100 g (Salim) to 11.62 mg/100 g (Dhahbi, Biskri, Mahmoudi). On average, free polyphenol levels were slightly higher under conventional farming (10.52 mg/100 g) than under organic farming (10.20 mg/100 g) for ferulic acid, and comparable for gallic acid (8.90 mg/100 g in conventional vs. 8.73 mg/100 g in organic). For free gallic acid, concentrations ranged from 7.47 mg/100 g (Monastir) to 10.27 mg/100 g (Saragolla) under organic farming, and from 7.57 mg/100 g (Salim) to 10.14 mg/100 g (Chili) under conventional farming. Mean values remained similar between the two systems (8.90 mg/100 g for conventional vs. 8.73 mg/100 g for organic). In contrast, the bound fraction showed more pronounced differences among varieties and much higher polyphenol levels. Bound ferulic acid ranged from 176.23 mg/100 g (Salim) to 335.42 mg/100 g (Biskri) under organic farming, and from 218.05 mg/100 g (Salim) to 339.98 mg/100 g (Dhahbi) under conventional farming. The overall mean was slightly higher in organic (283.91 mg/100 g) than in conventional farming (281.79 mg/100 g). Similarly, bound gallic acid showed variations ranging from 166.06 mg/100 g (Salim) to 288.45 mg/100 g (Chili) in organic farming and from 174.49 mg/100 g (Khiar) to 249.41 mg/100 g (Chili) in conventional farming. The organic average (213.62 mg/100 g) exceeded that of the conventional farming (205.32 mg/100 g), although extreme values reflected variety‐specific patterns.

In the present study, the local varieties Biskri, Mahmoudi, and Chili exhibited the highest free phenolic contents, whereas modern lines showed lower internal variability. Interestingly, among the modern varieties, Dhahbi displayed an exceptionally high TPC. Regarding total contents, ferulic acid ranged from 227.86 mg/100 g (Salim) to 347.46 mg/100 g (Biskri) under organic farming, and from 226.67 mg/100 g (Salim) to 351.60 mg/100 g (Dhahbi) under conventional farming. Global mean values showed a slight advantage for organic farming (294.11 mg/100 g) compared with conventional farming (292.30 mg/100 g). For total gallic acid, values ranged from 173.63 mg/100 g (Salim) to 298.48 mg/100 g (Chili) under organic farming, and from 173.63 mg/100 g (Salim) to 259.56 mg/100 g (Chili) under conventional conditions, with respective means of 222.36 mg/100 g (organic) and 214.22 mg/100 g (conventional). Overall, the variability observed among varieties and cultivation modes highlights both genetic and environmental influences on the biosynthesis of polyphenols in durum wheat. In the present study, local varieties exhibited the highest TPC levels, while modern varieties showed the lowest.

### 3.2 Identification of Phenolic Compounds in Durum Wheat Varieties

The identification of phenolic compounds in the free and bound extracts of durum wheat was confirmed by matching their retention times and UV–Visible spectra with those of their respective commercial pure standards. The phenolic acids identified and their distribution are presented in Table 3. Six phenolic acids were detected in most of the phenolic fractions of the organically grown wheat varieties, namely caffeic, sinapic, p‐coumaric, ferulic, diferulic (a, b, and c), and triferulic acids. In fact, six acids were identified exclusively in the bound phenolic fraction, whereas only one phenolic acid was detected in the free fraction. The phenolic acids identified in the bound fraction were caffeic, sinapic, p‐coumaric, ferulic, diferulic, and triferulic acids, while the only phenolic acid detected in the free fraction was p‐coumaric acid. Additional standards, including p‐hydroxybenzoic, syringic, and vanillic acids, were used to verify the presence of other phenolic acids. However, these compounds were not detected in the analyzed samples, most likely due to their occurrence at concentrations too low to be quantified using the applied analytical method.

**TABLE 3:** Mass spectral characteristics of phenolic compounds identified in durum wheat varieties.

Compound	R.T. (min)	[M‐H]^−^ (m/z)	MS^2^ ions (m/z)	MS^3^ ions (m/z)
Caffeic acid	3.29	179	136	
*p‐Coumaric acid*	4.36	163	119	
Ferulic acid	4.95	193	149, 177	134, 150
Sinapic acid	5.14	223	208, 178, 165	164, 180, 151
Diferulic acid a	5.17	385	**341**, 300, 250	326, 297, 283
Diferulic acid b	5.79	385	**340**, 298, 316, 249	297, 325, 282
Diferulic acid c	7.18	385	**341**, 327, 369, 282	326, 282, 298
Triferulic acid	8.10	577	533, 356, 311, 384	

*Note:* Fragment ions are listed in order of relative abundances; MS^2^ ions in bold were those subjected to MS^3^ fragmentation.

### 3.3 Phenolic Acid Quantification in Durum Wheat Varieties

The analysis of variance revealed that both farming system and variety exerted highly significant effects (*p* ≤ 0.001) on all bound and total fractions of the phenolic acids quantified in durum wheat varieties, reflecting a strong genotypic influence and marked sensitivity to growing conditions (Appendices S2 and S3). The combined effect of farming system and variety was also highly significant (*p* ≤ 0.001) for most bound and total fractions of the acids studied, indicating a differential genotypic response to farming systems; the only exception was the bound and total fractions of caffeic acid, for which this interaction was not significant.

In this context, caffeic acid was detected in the majority of durum wheat samples, mainly in the bound phenolic fractions, with the exception of the two modern varieties Salim and Monastir, in which it was entirely absent under both farming systems. No traces of caffeic acid were observed in the free fractions of any variety (Appendix S4). Under organic farming, caffeic acid content ranged from 2.89 mg/100 g (Razzak) to 3.13 mg/100 g (Jenah Khotifa). The local varieties Chili, Jenah Khotifa, Biskri, and Mahmoudi exhibited particularly high concentrations. Among the modern lines, Dhahbi, INRAT 100, and Khiar also showed relatively notable levels (3.10, 3.04, and 3.00 mg/100 g, respectively). Overall, the variety Chili consistently displayed the highest caffeic acid content across both farming systems, together with Dhahbi under conventional farming.

Sinapic acid concentrations ranged from 4.01 mg/100 g in the modern variety Salim to 4.47 mg/100 g in the local variety Biskri. This phenolic acid was detected in all genotypes examined except the modern variety Khiar, where it was absent under both systems. Among local varieties, Biskri and Mahmoudi showed the highest levels (4.47 and 4.38 mg/100 g, respectively). Several modern lines, notably Razzak and Maali, also exhibited relatively high sinapic acid concentrations (4.40 and 4.32 mg/100 g, respectively). On average, organic farming induced a slightly higher sinapic acid content (4.29 mg/100 g) compared with conventional farming (4.13 mg/100 g) (Appendix S4).

p‐Coumaric acid was the only phenolic acid present in both free and bound fractions. As expected, bound p‐coumaric acid concentrations were higher than those of the free fraction under both farming systems. In organic culture, free p‐coumaric acid ranged from 0.75 mg/100 g (Khiar) to 1.20 mg/100 g (Razzak), whereas bound p‐coumaric acid ranged from 3.96 mg/100 g (Khiar) to 5.01 mg/100 g (Biskri). Statistically significant differences were observed between samples from the two farming systems. Consequently, total p‐coumaric acid (sum of free and bound fractions) was higher under organic farming (5.45 mg/100 g) than under conventional farming (4.77 mg/100 g) (Appendix S4).

Ferulic acid content ranged from 16.27 to 73.87 mg/100 g, reflecting substantial variability between genotypes and farming systems (Appendix S4). On average, organic farming showed a higher concentration (44.87 mg/100 g) than conventional farming (36.76 mg/100 g). Among improved lines, Dhahbi, Razzak, and Saragolla stood out with high ferulic acid levels (57.10, 56.11, and 46.51 mg/100 g in organic, respectively), whereas Khiar and Maali exhibited the lowest values (16.27 and 21.00 mg/100 g, respectively). Among local varieties, Biskri showed the highest content (73.87 mg/100 g), followed by Chili (60.46 mg/100 g), Mahmoudi (54.97 mg/100 g), and Jenah Khotifa (51.49 mg/100 g).

Diferulic acid a was detected only in the bound fraction and exhibited concentrations that varied with variety and farming system (Appendix S5). Mean levels were higher in organic farming (19.38 mg/100 g) than in conventional farming (15.80 mg/100 g). Among improved lines, the highest values under organic farming were recorded for Dhahbi (26.76 mg/100 g), Saragolla (23.48 mg/100 g) and Razzak (17.72 mg/100 g); the lowest values were observed in Khiar (10.87 mg/100 g) and Karim (10.08 mg/100 g), while Salim showed a non‐quantifiable level. For the local varieties, all exhibited relatively high concentrations under organic farming, ranging from 20.95 mg/100 g (Mahmoudi) to 28.43 mg/100 g (Jenah Khotifa), the latter representing the highest value among all genotypes. Chili (25.50 mg/100 g) and Biskri (24.57 mg/100 g) also had substantial concentrations, exceeding those of most improved varieties.

Diferulic acid b was detected in all durum wheat varieties, with values varying by farming system. Values ranged from 20.36 to 125.40 mg/100 g, revealing considerable inter‐varietal variability (Appendix S5). On average, organic farming recorded a higher content (68.91 mg/100 g) than conventional farming (56.34 mg/100 g). Among genotypes, the local varieties Biskri and Jenah Khotifa showed the highest values (125.40 and 125.17 mg/100 g, respectively), followed by Chili (98.12 mg/100 g) and Mahmoudi (97.77 mg/100 g). Conversely, the modern lines Salim and Khiar exhibited the lowest concentrations (20.36 and 31.79 mg/100 g, respectively).

Diferulic acid c represented the most abundant dimeric form among ferulic acids identified in the durum wheat varieties (Appendix S5). Concentrations varied widely according to farming system: under organic farming they ranged from 80.18 mg/100 g (Salim) to 348.97 mg/100 g (Biskri), whereas under conventional farming they varied from 59.50 mg/100 g (Salim) to 267.91 mg/100 g (Dhahbi). Overall, organic farming was characterized by a higher mean concentration (197.89 mg/100 g) than conventional farming (136.54 mg/100 g). Among local varieties, Biskri and Chili exhibited the highest values (348.97 and 334.53 mg/100 g, respectively), followed by Jenah Khotifa (284.88 mg/100 g). Among improved lines, Dhahbi and Maali also showed high levels (284.47 and 224.99 mg/100 g, respectively). In contrast, Salim and Monastir displayed the lowest concentrations (80.18 and 82.32 mg/100 g, respectively).

Triferulic acid concentrations also varied notably with variety and farming system. The highest values were observed in Chili and Jenah Khotifa under organic farming (41.39 and 41.05 mg/100 g, respectively), while the lowest were found in Salim (8.69 mg/100 g in conventional and 10.73 mg/100 g in organic). Overall, organic farming favored a significant increase in triferulic acid levels, with a mean of 31.33 mg/100 g versus 21.17 mg/100 g in conventional farming (Appendix S5).

**FIGURE 1:**
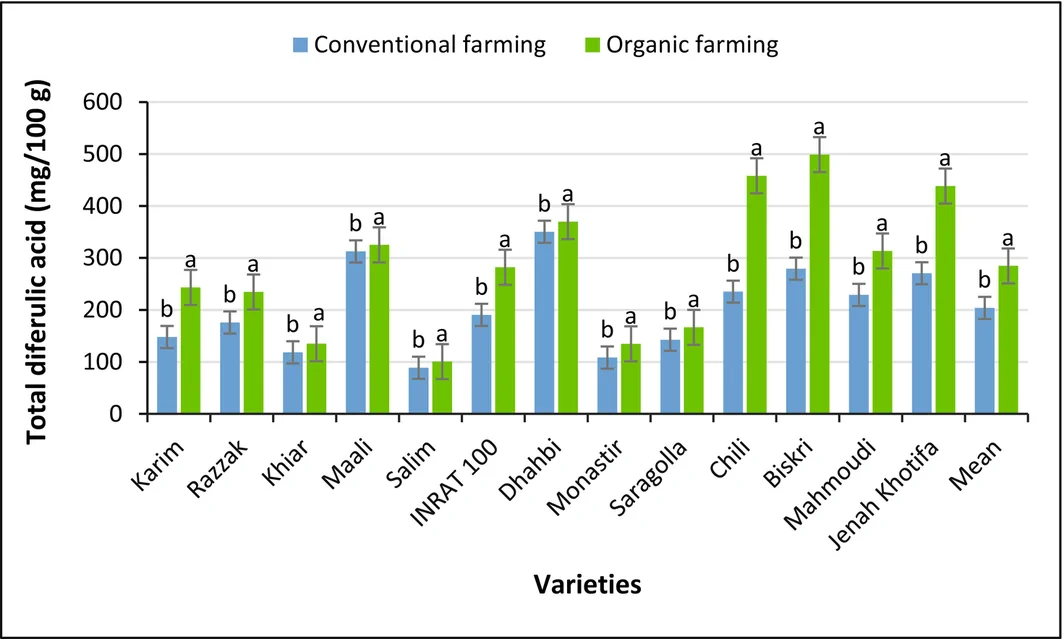
Total diferulic acid content (mg/100 g) of durum wheat genotypes grown under conventional and organic farming systems. Different letters within the same row indicate statistically significant differences (*p* ≤ 0.05, Tukey's test).

Total diferulic acid analysis revealed pronounced differences among varieties and farming systems (Figure 1; Appendices S6 and S7). The highest concentrations were recorded in Biskri and Chili under organic farming (498.94 and 458.15 mg/100 g, respectively), whereas Salim showed the lowest levels (88.68 mg/100 g in conventional and 100.54 mg/100 g in organic). These data clearly indicate that organic farming tends to stimulate accumulation of diferulic acids, with the overall mean increasing from 203.82 mg/100 g in conventional systems to 284.69 mg/100 g in organic systems (Appendix S7).

**FIGURE 2:**
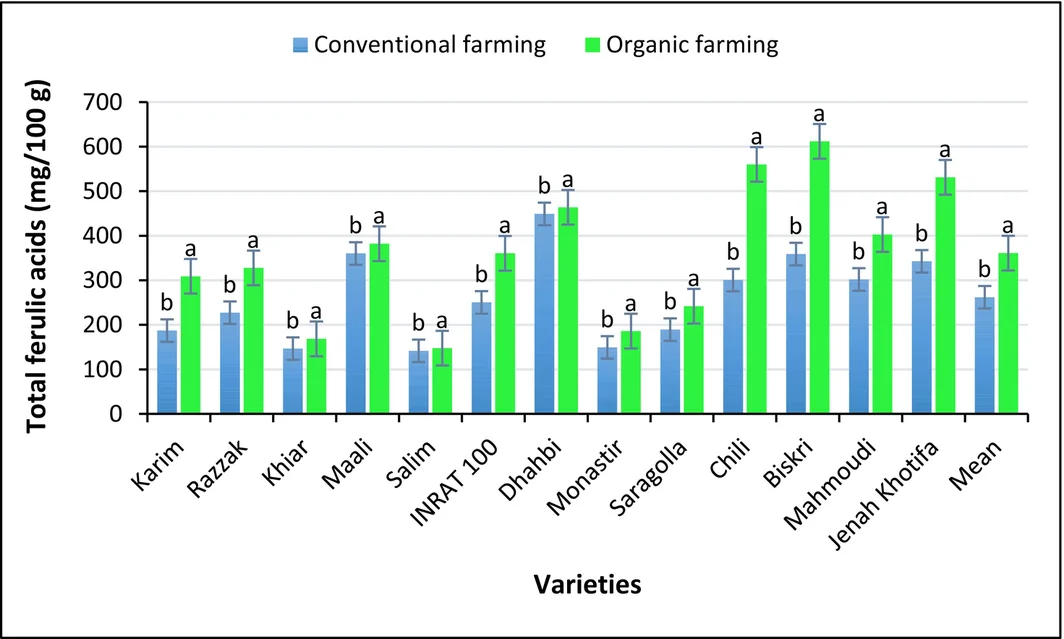
Total ferulic acid content (mg/100 g) of durum wheat genotypes grown under conventional and organic farming systems. Different letters within the same row indicate statistically significant differences (*p* ≤ 0.05, Tukey's test).

The sum of ferulic acids (including forms a, b, c, triferulic, and monomeric ferulic acid) showed marked differences by variety and farming system (Figure 2; Appendices S6 and S7). The highest totals were observed in Biskri and Chili under organic farming (611.85 and 560.00 mg/100 g, respectively), whereas Salim displayed the lowest totals (141.37 mg/100 g in conventional and 147.56 mg/100 g in organic). Overall, organic farming promoted higher accumulation of these compounds, with the mean rising from 261.75 mg/100 g in conventional to 360.89 mg/100 g in organic systems (Appendix S7). In general, organic farming was associated with higher phenolic compound concentrations than conventional farming. Moreover, local varieties exhibited higher concentrations for most acids compared with modern varieties. By contrast, the variety Salim was characterized by generally lower values for almost all phenolic acids, emphasizing its limited phenolic profile.

### 3.4 Antioxidant Activity of Durum Wheat Varieties

The antioxidant activity of the free, bound, and total phenolic fractions of durum wheat samples was determined using the Ferric Reducing Antioxidant Power assay (FRAP) (Table 4). This assay revealed substantial variability among the different durum wheat varieties and between farming systems. As shown in Table 4, a highly significant variation (*p* ≤ 0.001) in antioxidant activity was detected between farming systems, between varieties, and for their interaction, in free, bound, and total phenolic fractions (Appendix S6).

**TABLE 4:** FRAP values (mmol Fe^2+^/100 g) of durum wheat varieties.

Varieties	Farming system	Free	Bound	Total
Karim	CF	0.16 ± 0.02^a^	2.57 ± 0.15^b^	2.73 ± 0.15^b^
	OF	0.13 ± 0.00^b^	2.84 ± 0.03^a^	2.97 ± 0.03^a^
Razzak	CF	0.11 ± 0.01^b^	2.73 ± 0.02^b^	2.84 ± 0.02^b^
	OF	0.17 ± 0.00^a^	2.98 ± 0.03^a^	3.15 ± 0.04^a^
Khiar	CF	0.16 ± 0.03^a^	2.45 ± 0.01^a^	2.61 ± 0.01^a^
	OF	0.16 ± 0.01^a^	2.15 ± 0.25^b^	2.31 ± 0.25^b^
Maali	CF	0.17 ± 0.00^b^	2.37 ± 0.06^b^	2.54 ± 0.07^b^
	OF	0.19 ± 0.00^a^	2.65 ± 0.03^a^	2.84 ± 0.03^a^
Salim	CF	0.14 ± 0.01^a^	2.19 ± 0.06^a^	2.33 ± 0.06^a^
	OF	0.14 ± 0.00^a^	1.89 ± 0.13^b^	2.03 ± 0.12^b^
INRAT 100	CF	0.15 ± 0.01^a^	2.50 ± 0.15^b^	2.65 ± 0.16^b^
	OF	0.12 ± 0.02^b^	2.82 ± 0.04^a^	2.94 ± 0.05^a^
Dhahbi	CF	0.20 ± 0.01^a^	2.63 ± 0.05^b^	2.83 ± 0.05^b^
	OF	0.15 ± 0.00^b^	3.07 ± 0.02^a^	3.22 ± 0.01^a^
Monastir	CF	0.16 ± 0.02^a^	2.04 ± 0.02^a^	2.20 ± 0.02^a^
	OF	0.16 ± 0.04^a^	1.96 ± 0.16^b^	2.13 ± 0.17^b^
Saragolla	CF	0.10 ± 0.00^b^	2.30 ± 0.02^b^	2.40 ± 0.02^b^
	OF	0.16 ± 0.03^a^	2.37 ± 0.02^a^	2.52 ± 0.03^a^
Chili	CF	0.20 ± 0.00^b^	2.85 ± 0.01^b^	3.04 ± 0.01^b^
	OF	0.25 ± 0.00^a^	3.13 ± 0.02^a^	3.38 ± 0.02^a^
Biskri	CF	0.14 ± 0.01^b^	2.86 ± 0.02^b^	3.00 ± 0.01^b^
	OF	0.21 ± 0.00^a^	3.09 ± 0.11^a^	3.29 ± 0.11^a^
Mahmoudi	CF	0.13 ± 0.00^b^	2.82 ± 0.03^b^	2.95 ± 0.03^b^
	OF	0.18 ± 0.02^a^	3.08 ± 0.06^a^	3.26 ± 0.07^a^
Jenah Khotifa	CF	0.18 ± 0.00^b^	2.81 ± 0.05^b^	2.99 ± 0.04^b^
	OF	0.22 ± 0.00^a^	3.43 ± 0.02^a^	3.65 ± 0.02^a^
Mean	CF	**0.15 ± 0.03** ^b^	**2.55 ± 0.27** ^b^	**2.70 ± 0.27** ^b^
	OF	**0.17 ± 0.04** ^a^	**2.73 ± 0.48** ^a^	**2.90 ± 0.50** ^a^
*ANOVA*				
Farming system		***	***	***
Variety		***	***	***
FS × variety		***	***	***

*Note:* *** indicates a significant effect at the 0.1% probability level. Values are expressed as mean ± standard deviation (n=3). Different letters within the same row indicate statistically significant differences (*p* ≤ 0.05, Tukey’s test). CF: conventional farming; OF: organic farming. The bold values correspond to the mean values calculated across all varieties for each of the two farming systems, namely Conventional Farming (CF) and Organic Farming (OF).

In organic farming, the free phenolic fraction ranged from 0.12 mmol Fe/100 g (INRAT 100) to 0.25 mmol Fe/100 g (Chili), while under conventional farming, values ranged from 0.10 mmol Fe/100 g (Saragolla) to 0.20 mmol Fe/100 g (Dhahbi and Chili). For the bound fraction, concentrations ranged from 1.89 mmol Fe/100 g (Salim) to 3.43 mmol Fe/100 g (Jenah Khotifa) in organic farming, whereas under conventional farming, they varied from 2.04 mmol Fe/100 g (Monastir) to 2.86 mmol Fe/100 g (Biskri).

On average, the bound phenolic fraction exhibited markedly higher FRAP values than the free fraction. Overall, organic farming exhibited higher antioxidant activity, with mean values of 0.17 mmol Fe/100 g (free fraction) and 2.73 mmol Fe/100 g (bound fraction), compared with 0.15 and 2.55 mmol Fe/100 g under conventional farming. Among the improved lines, the highest values were recorded in Dhahbi (3.07 mmol Fe/100 g in organic farming), Razzak (2.98 mmol Fe/100 g) and INRAT 100 (2.82 mmol Fe/100 g), whereas Salim and Monastir showed the lowest levels (1.89 and 1.96 mmol Fe/100 g, respectively). Regarding local varieties, all exhibited particularly high antioxidant activity. Jenah Khotifa showed the highest value (3.43 mmol Fe/100 g in organic farming), followed by Chili (3.13 mg/100 g), Biskri (3.09 mg/100 g) and Mahmoudi (3.08 mg/100 g). These varieties also showed the highest total phenolic content (TPC), reaching 3.65 mg/g for Jenah Khotifa and 3.38 mg/g for Chili in organic farming, whereas under conventional farming, the highest value was 3.04 mg/g for Chili. The mean TPC of durum wheat varieties was 2.70 mg/g in conventional farming and 2.90 mg/g in organic farming. Overall, local varieties, particularly Chili, Jenah Khotifa and Biskri, exhibited relatively high FRAP values, confirming a strong association between phenolic content and antioxidant activity.

### 3.5 Overall Assessment of Phenolic Biodiversity Using Principal Component Analysis and Clustering Analysis

**FIGURE 3:**
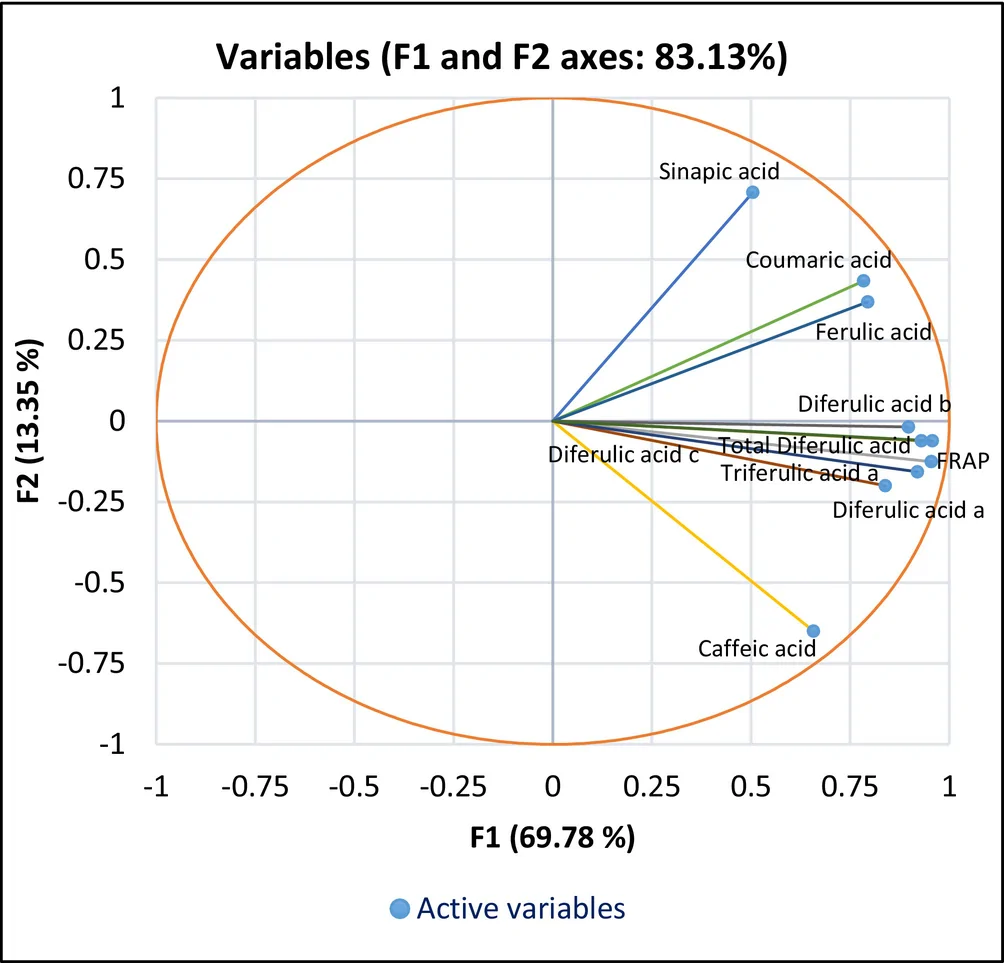
Principal component analysis.

Principal component analysis (PCA) conducted on the 13 durum wheat varieties under organic farming explained 83.13% of the total variance (Figure 3).

The loading plot for these principal components (PCs), presented in Figure 3, indicates that the first axis (PC1), accounting for 69.78% of the variance, reflects the gradient of phenolic richness and antioxidant capacity (FRAP), with strong positive correlations for ferulic, p‐coumaric, and sinapic acids, whereas caffeic acid primarily contributes to the second axis (PC2), explaining 13.35% of the variance. Varieties with high concentrations of these bound phenolics also exhibited elevated FRAP values, confirming the link between phenolic content and antioxidant activity.

**FIGURE 4:**
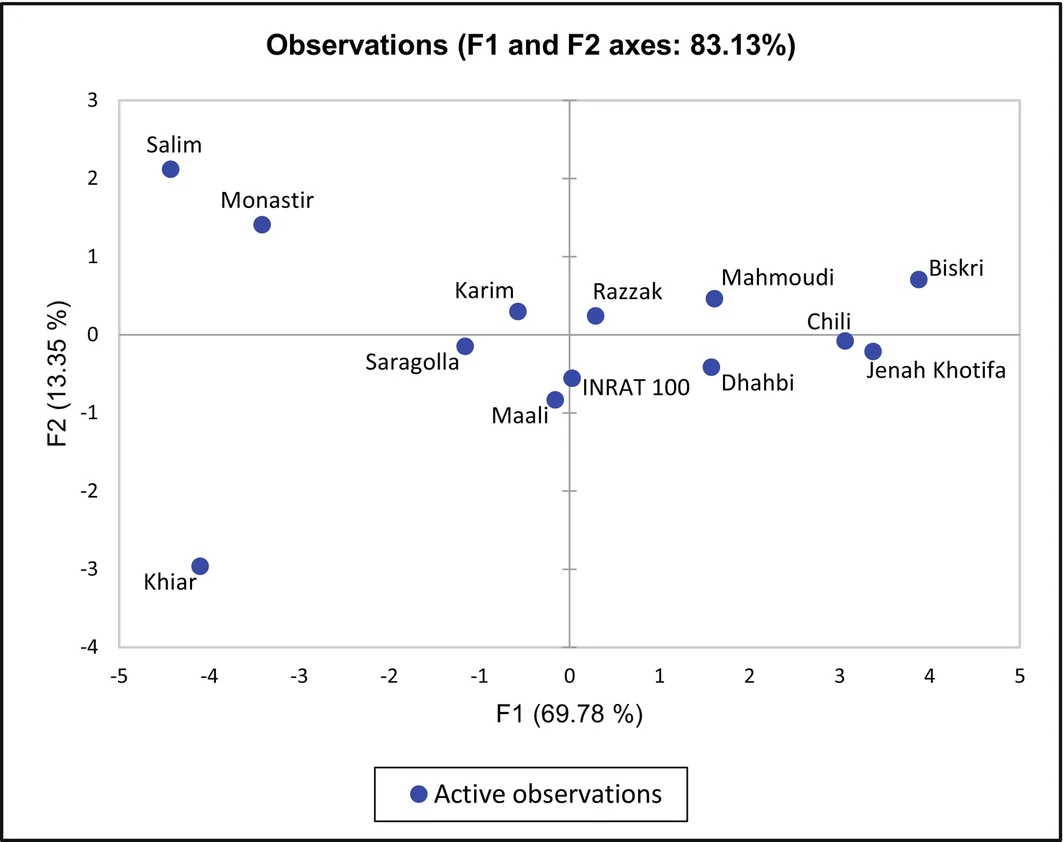
Factor scores of organic durum wheat samples projected onto the first two principal components after rotation.

The projection of scores for the 13 durum wheat varieties on the consensus space defined by PC1 and PC2 is shown in Figure 4. The PC1 axis (69.78%), representing the majority of the variance, primarily separates varieties based on total phenolic content and overall antioxidant capacity (FRAP): Biskri, Jenah Khotifa, Chili, Dhahbi, Mahmoudi, and Razzak, positioned on the right, displayed high phenolic content and antioxidant power, whereas Salim, Monastir, Khiar, and Saragolla, positioned on the left, exhibited lower levels.

PC2 (13.35% of variance) highlights qualitative differences in phenolic profiles: Salim and Monastir, strongly positive on PC2, were characterized by elevated concentrations of specific phenolic compounds, whereas Khiar, highly negative, showed a deficient profile in these compounds. Karim was slightly positive, indicating a moderately favorable profile for certain phenolics, while Maali, INRAT 100, and Saragolla, near the origin on PC2, exhibited intermediate phenolic composition and antioxidant activity.

**FIGURE 5:**
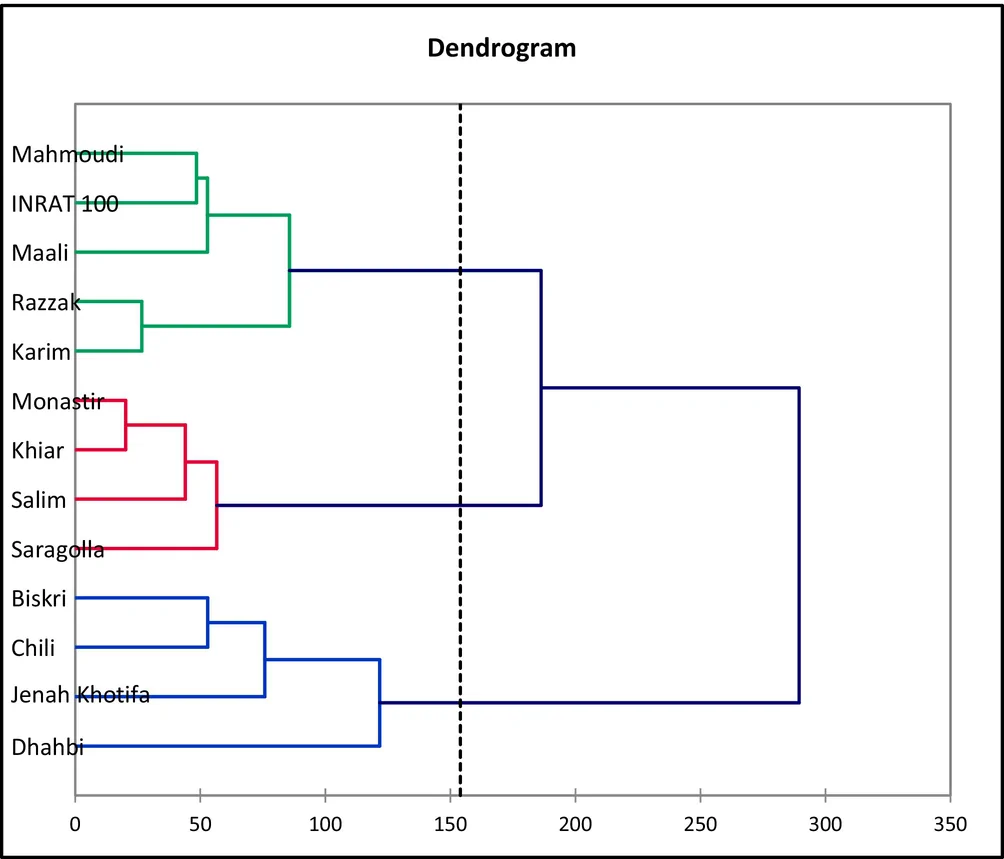
Dendrogram of durum wheat varieties and overall assessment of polyphenol biodiversity.

To further support these observations, hierarchical clustering analysis was performed (Figure 5), revealing the formation of three main clusters.

The first cluster, subdivided into two subgroups, included the local variety Mahmoudi and two improved varieties (INRAT 100 and Maali), and the modern varieties Razzak and Karim. These varieties exhibited moderate phenolic content and intermediate antioxidant activity. The second cluster comprised the improved varieties Monastir, Khiar, Salim, and Saragolla, which displayed the lowest concentrations of phenolic compounds and reduced antioxidant capacity. Finally, the third cluster grouped three local varieties (Biskri, Chili, and Jenah Khotifa) along with a single modern line (Dhahbi), which were distinguished by high levels of phenolic compounds and strong antioxidant activity.

## 4 Discussion

The free, bound, and total polyphenol contents of durum wheat varieties revealed that a moderate reduction in the synthesis of free polyphenols under organic conditions is possibly linked to lower nitrogen availability or differential regulation of phenylpropanoid pathways. Moreover, the contribution of this acid to the free fraction is relatively stable regardless of farming system, while these observations confirm that bound phenolics represent the main phenolic pool in wheat grain. Indeed, these findings suggest a potential activation of phenolic binding mechanisms to the cell wall under organic stress conditions, with the bound fraction richer than the free fraction. Free polyphenols generally represent the smallest contribution (typically < 0.5%–1% percent) to the total polyphenol pool in cereals, consistent with Adom and Liu (2002), who showed that bound phenolics account for approximately 77% of the total polyphenols. Moreover, according to Higuchi (2014), most phenolic acids in wheat grain are insoluble and covalently attached to cell wall polysaccharides, such as arabinoxylans and lignin, through ester ether linkages. In addition, several studies have shown that cereals produced under organic farming systems often exhibit higher concentrations of secondary metabolites, particularly phenolic compounds, due to greater physiological stress (Barański et al. 2014), which typically results from intensified biotic and abiotic pressures and reduced nitrogen availability stimulating the biosynthesis of defensive secondary metabolites (Konopka et al. 2012). Therefore, organic farming may enhance the accumulation of phenolic compounds, likely in response to increased abiotic stress associated with lower chemical inputs (Augšpole et al. 2021).

These findings underscore that bound compounds, which constitute the major fraction of phenolic acids in durum wheat grain, are the most responsive to genetic and environmental variation, whereas free forms, limited here to p‐coumaric acid, are minor and less responsive, indicating an overall greater accumulation of this acid in organic conditions. Ferulic acid was the predominant phenolic acid identified across the studied durum wheat varieties, in agreement with previous reports (Boukid et al. 2019; Okarter et al. 2010), and is mainly abundant in the aleurone layer and bound to cell‐wall components (e.g., arabinoxylans) through ester and ether linkages (Dinelli et al. 2011). These observations suggest that organic conditions favor accumulation of this acid and also promote formation of cross‐linking diferulic acids. Diferulic acid a content is strongly influenced by genotype and farming system, with a general tendency for greater accumulation under organic farming, particularly in local varieties, indicating enhanced accumulation under the organic system. Local varieties possess a greater capacity to accumulate diferulic acid b, likely related to genetic differences in cell‐wall composition and structure, reflecting the combined effect of genotype and farming system and suggesting a probable stimulation of di‐ether bond formation within cell walls under organic conditions. Diferulic acid c is a relevant marker of cell‐wall stiffening, whose biosynthesis appears to be promoted under organic farming, supporting the role of bound phenolics in structural reinforcement. These observations align with recent studies reporting that organic systems tend to increase phenolic compound contents relative to conventional systems, likely in response to altered abiotic/biotic stresses and fertilization practices (Giampieri et al. 2022; Kowalska et al. 2025). Furthermore, the commonly observed superiority of local varieties in phenolic content has been reported in several comparative studies, suggesting that genotypes not selected for maximum yield maintain higher metabolic capacity (Györéné Kis et al. 2025; Rachoń et al. 2025). The importance of genotype × environment (G × E) interactions in explaining variability is supported by recent research on durum wheat and other cereals, showing that G × E can outweigh isolated effects of genotype or farming system (Melios et al. 2024). Observed differences among varieties and farming systems may also be attributed to differential regulation of metabolic pathways involved in phenolic acid biosynthesis. Ma et al. (2022) reported that environmental conditions and nutrient availability influence the expression of genes encoding key enzymes of these pathways, notably phenylalanine ammonia‐lyase (PAL), and Giampieri et al. (2022) demonstrated that organic farming enhances activation of these defense mechanisms, leading to greater accumulation of phenolic compounds. The predominance of ferulic acid among wheat phenolics reflects its major role in cell‐wall structure and contribution to grain antioxidant potential (Rachoń et al. 2025). Overall, moderate stresses under organic farming stimulate secondary metabolite biosynthesis, strengthening the overall antioxidant profile of wheat.

The FRAP method evaluates the ability of antioxidants to reduce ferric ions (Fe^3+^) to ferrous ions (Fe^2+^), reflecting the presence of electron‐donating antioxidant compounds (Leoncini et al. 2012). These results indicate a strong dependence of antioxidant activity on both genotype and farming system, associated with the qualitative and quantitative diversity of phenolic compounds present (Vaher et al. 2010). Bound phenolics represent the major contributor to antioxidant activity in durum wheat grain, confirming their predominance in the overall reducing capacity of the grain. Organic farming, which exposes plants to moderate oxidative stress, promotes biosynthesis of phenolic metabolites responsible for antioxidant activity, confirming their richness in phenolic compounds and indicating a slight increase in total phenolics under organic systems. Antioxidant activity in durum wheat depends on both genotype and farming system, with a clear tendency toward higher reducing capacity under organic farming, particularly in local varieties, in agreement with Żuchowski et al. (2009, 2011). Most phenolics in wheat grain are concentrated in the cell wall, associated with polysaccharides and lignans, and partially lost during milling and flour refinement (Vaher et al. 2010). Heterogeneity among varieties can be mainly attributed to the qualitative and quantitative diversity of phenolic compounds in the grains, primarily in bound form concentrated in the aleurone layer, seed coat, and embryo Kosík et al. (2014), which play a key role in reducing ferric ions. The phenolic composition is strongly influenced by genetics, pedoclimatic conditions, and agronomic practices (Laddomada et al. 2021). Bound phenolic fractions contribute more significantly to total antioxidant activity than free fractions (Okarter et al. 2010; Žilić et al. 2012), and may exert prolonged protective effects in the intestine. Variability among varieties also reflects differences in ferulic, p‐coumaric, and vanillic acids, major contributors to grain antioxidant capacity (Liyana‐Pathirana et al. 2006). Local varieties, particularly Chili, Jenah Khotifa, and Biskri, exhibited high FRAP values, suggesting natural richness in bioactive compounds due to limited genetic selection and adaptation to local conditions (Boukid et al. 2019), often exceeding modern improved lines (Giacosa et al. 2022). Consequently, the increased antioxidant activity under organic farming is relevant for functional food applications, enhancing nutritional potential and supporting human health (de Oliveira et al. 2017; Melios et al. 2024). The strong correlation between total phenolic content and FRAP values confirms that phenolic compounds are the main contributors to antioxidant activity and associated health‐promoting properties (Di Loreto et al. 2018), reinforcing the importance of selecting local varieties with high antioxidant capacity for functional foods. Beyond nutritional quality, agronomic performance is an important criterion for evaluating the potential of durum wheat genotypes under sustainable organic farming systems. In a complementary study on the same thirteen Tunisian durum wheat varieties, Boukrain, Martínez‐Villaluenga, et al. (2026) showed that organic farming may reduce grain yield compared with conventional management; however, several genotypes maintained acceptable productivity under low‐input Mediterranean conditions. This balance between agronomic adaptation and enhanced bioactive potential is particularly relevant, as genotype and environment strongly influence phenolic accumulation and antioxidant capacity in durum wheat (Melios et al. 2024). Similarly, Carucci et al. (2023) emphasized the importance of selecting adapted genotypes capable of combining acceptable productivity and grain quality under organic farming conditions. Therefore, the local varieties exhibiting high phenolic content and antioxidant capacity may represent valuable genetic resources for sustainable durum wheat production systems.

The high phenolic content and antioxidant capacity observed in Tunisian durum wheat landraces are consistent with previous findings reported for traditional Mediterranean wheat genetic resources. Studies conducted on indigenous Turkish wheat varieties demonstrated substantial variability in phenolic composition and antioxidant potential among genotypes, with multivariate approaches such as PCA effectively discriminating varieties according to their bioactive profiles (Akram et al. 2025). Similarly, investigations on ancient and modern durum wheat genotypes have revealed significant differences in phenolic profiles among genotypes, highlighting the potential value of traditional varieties as sources of bioactive compounds (Di Loreto et al. 2018). Other studies have also shown that ancient wheat varieties may retain valuable biochemical characteristics associated with enhanced phenolic diversity and antioxidant potential (Giacosa et al. 2022). These findings support the idea that Mediterranean landraces represent valuable reservoirs of functional compounds and adaptive traits. Therefore, the Tunisian landraces identified in the present study, particularly Biskri, Chili, and Jenah Khotifa, contribute to the broader Mediterranean effort to conserve and exploit ancient genetic resources for sustainable agriculture and the development of nutritionally valuable wheat products.

Principal component analysis (PCA) and clustering analysis further support these observations. PCA results show that organic farming stimulates polyphenol biosynthesis through biotic and abiotic stress mechanisms, enhancing grain antioxidant potential (Martín‐Diana et al. 2021). The phenolic profile serves as a reliable indicator of overall antioxidant activity in cereals (Kwiatkowski et al. 2022), suggesting that varieties such as Biskri, Jenah Khotifa, and Chili could be prioritized for nutritional applications, whereas Salim and Monastir may be targeted for extraction of specific phenolics. In this context, PCA allows identification of quantitative and qualitative differences among varieties, providing insights for selection and breeding of durum wheat for functional purposes (Cheynier et al. 2013; Marcotuli et al. 2025). This structuring confirms that genetic variability reflects intrinsic metabolic and functional differences, not solely age or breeding status. Overall, these findings provide valuable information for selecting high‐functional organic durum wheat varieties and exploiting phenolic compounds in nutritional and technological applications (Laddomada et al. 2015; Marcotuli et al. 2025). Although the present study provides valuable insights into the effects of organic and conventional farming systems on the phenolic composition and antioxidant capacity of Tunisian durum wheat varieties, it should be acknowledged that the experiment was conducted during a single growing season (2023–2024). Consequently, the observed responses reflect the specific environmental conditions prevailing during that cropping season and should therefore be interpreted within this climatic context. Nevertheless, because all 13 durum wheat genotypes were cultivated simultaneously under identical agronomic management practices, soil conditions, and experimental design, the comparisons between farming systems and genotypes remain scientifically robust and reliable. Therefore, the present study provides a sound assessment of the specific influence of farming systems on phenolic accumulation and antioxidant capacity under homogeneous environmental conditions. However, further multi‐year and multi‐environment studies are needed to assess the stability of these traits under contrasting climatic conditions and to better characterize genotype × environment interactions. Such investigations will contribute to a more comprehensive understanding of the long‐term response of Tunisian durum wheat germplasm to different farming systems and environmental conditions.

## 5 Conclusions

This investigation revealed significant variability among the studied genotypes, confirming the richness of genetic diversity in Tunisian durum wheat. Overall, organic farming led to higher total polyphenol content and enhanced antioxidant capacity (FRAP), suggesting that organic farming conditions promote the accumulation of beneficial secondary metabolites. UHPLC–MS^n^ analyses enabled the identification of eight phenolic acids; as expected, the majority of quantified acids were present in the bound form, while free forms were minor. Among the tested varieties, certain landraces, notably Chili, Biskri, and Jenah Khotifa, stood out for their high phenolic content and strong antioxidant potential under organic cultivation, positioning them as promising resources for breeding programs focused on nutritional and functional quality. Principal component analysis (PCA) and clustering analyses applied to the phenolic profiles confirmed that genotype differentiation is primarily driven by phenolic composition and highlighted groups of varieties sharing common phenolic signatures. These analyses also identified specific varieties (landraces such as Chili, Biskri, Jenah Khotifa, and the modern cultivar Dhahbi) as priority candidates for targeted valorization under organic systems. These findings emphasize the agronomic and nutritional value of local Tunisian durum wheat varieties and support the integration of organic farming into sustainable production strategies. However, since this study was conducted during a single growing season (2023–2024), these findings should be interpreted within the environmental conditions evaluated. Further multi‐year and multi‐environment studies are needed to confirm the stability of these responses under different climatic conditions. They pave the way for the exploitation of genotypes adapted to Mediterranean agroecological conditions and meeting the growing demand for foods with high health‐promoting properties.

## Author Contributions

**Anis Boukrain:** conceptualization, methodology, software, resources, project administration, writing – original draft, writing – review and editing, funding acquisition, investigation, formal analysis, data curation. **Chirine Brinsi:** supervision, visualization, validation, software. **Mondher Mejri:** visualization, writing – review and editing, supervision, validation, funding acquisition, project administration.

## Conflicts of Interest

The authors declare no conflicts of interest.

## Supporting information


**Appendix S1:** Analysis of variance of total, free, and bound polyphenol contents of thirteen durum wheat varieties under two farming systems.
**Appendix S2:** Mean squares for the quantification of phenolic acids and antioxidant capacity in thirteen durum wheat varieties under two farming systems.
**Appendix S3:** Mean squares for the quantification of phenolic acids and antioxidant capacity in thirteen durum wheat varieties under two farming systems (continued).
**Appendix S4:** Phenolic profile of thirteen durum wheat varieties grown under organic and conventional farming (mg/100 g) *(nd: not detected, nq: not quantified)*.
**Appendix S5:** Phenolic profile of thirteen durum wheat varieties grown under organic and conventional farming (mg/100 g).
**Appendix S6:** Mean squares for the quantification of phenolic acids and antioxidant capacity in thirteen durum wheat varieties under two farming systems.
**Appendix S7:** Phytochemical Profile of durum wheat genotypes.

## Data Availability

The data generated and analyzed in this study are available in the Supporting Information.
