# Supplementary material for: Evaluation of the Phenolic Profile and Antioxidant Capacity of Landraces and Modern Tunisian Durum Wheat Genotypes Under Organic Farming System
Source: Food Sci Nutr. 2026 Sep 23;14(9):e72356. doi: 10.1002/fsn3.72356 (PMC13598799; doi:10.1002/fsn3.72356)
Supplement: Supplementary file 1 [file FSN3-14-e72356-s001.docx]

**Appendix 1 |**

Analysis of variance of total, free, and bound polyphenol contents of thirteen durum wheat varieties under two farming systems.

|  |  | **mg ferulic acid / 100 g of grain** | | | **mg gallic acid / 100 g of grain** | | |
| --- | --- | --- | --- | --- | --- | --- | --- |
| **Source of variation** | **df** | **Free** | **Bound** | **Total** | **Free** | **Bound** | **Total** |
| Corrected Model | 27 | 2.90 | 2552.00 | 2708.22 | 1.83 | 3365.99 | 3507.01 |
| Constant | 1 | 8367.98 | 6240236.70 | 6705607.15 | 6064.28 | 3422480.06 | 3716753.98 |
| Replication | 2 | 0.00 | 139.77 | 140.62 | 0.01 | 10.77 | 10.75 |
| Farming system | 1 | 1.95^***^ | 88.09^***^ | 63.81^***^ | 0.55^***^ | 1343.94^***^ | 1289.38^***^ |
| Variety | 12 | 5.78^ns^ | 4952.12^***^ | 5267.12^***^ | 3.48^***^ | 5973.37^***^ | 6251.83^***^ |
| Farming system * Variety | 12 | 0.58^ns^ | 759.25^***^ | 797.62^***^ | 0.59^***^ | 1486.31^***^ | 1529.70^***^ |
| Error | 50 | 0.02 | 71.36 | 72.90 | 0.00 | 12.68 | 12.87 |
| Total | 78 |  |  |  |  |  |  |
| Corrected Total | 77 |  |  |  |  |  |  |
| **CV** |  | **6.10** | **10.91** | **10.84** | **6.24** | **16.45** | **16.11** |
| **R^2^** |  | **0.98** | **0.95** | **0.95** | **1.00** | **0.99** | **0.99** |
| *ns and *** indicate a non-significant effect and a significant effect at the 0.1% probability level, respectively.* | | | | | | | |

**Appendix 2 |**

Mean squares for the quantification of phenolic acids and antioxidant capacity in thirteen durum wheat varieties under two farming systems.

|  |  | **Caffeic acid** | | **Sinapic acid** | | **Coumaric acid** | | | **Ferulic acid** | |
| --- | --- | --- | --- | --- | --- | --- | --- | --- | --- | --- |
| **Source of variation** | **df** | **Bound** | **Total** | **Bound** | **Total** | **Bound** | **Free** | **Total** | **Bound** | **Total** |
| Corrected Model | 27 | 0.026 | 0.026 | 0.04 | 0.04 | 0.44 | 0.17 | 0.85 | 661.71 | 661.71 |
| Constant | 1 | 580.40 | 580.40 | 1275.46 | 1275.46 | 1380.46 | 63.67 | 2037.05 | 129946.39 | 129946.39 |
| Replication | 2 | 0.002 | 0.002 | 0.01 | 0.01 | 0.02 | 0.01 | 0.01 | 11.87 | 11.87 |
| Farming system | 1 | 0.203^***^ | 0.203^***^ | 0.43^***^ | 0.43^***^ | 4.22^***^ | 0.88^***^ | 8.94^***^ | 1281.83^***^ | 1281.83^***^ |
| Variety | 12 | 0.034^***^ | 0.034^***^ | 0.03^***^ | 0.03^***^ | 0.47^***^ | 0.21^***^ | 0.95^***^ | 1149.16^***^ | 1149.16^***^ |
| Farming system * Variety | 12 | 0.005^ns^ | 0.005^ns^ | 0.03^***^ | 0.03^***^ | 0.17^***^ | 0.09^***^ | 0.22^***^ | 230.88^***^ | 230.88^***^ |
| Error | 50 | 0.002 | 0.002 | 0.00 | 0.00 | 0.02 | 0.00 | 0.02 | 10.20 | 10.20 |
| Total | 78 |  |  |  |  |  |  |  |  |  |
| Corrected Total | 77 |  |  |  |  |  |  |  |  |  |
| **CV** |  | **2.95** | **2.95** | **2.48** | **2.48** | **7.99** | **17.06** | **8.78** | **36.65** | **36.65** |
| **R^2^** |  | **0.85** | **0.85** | **0.90** | **0.90** | **0.93** | **0.95** | **0.95** | **0.97** | **0.97** |
| *ns and *** indicate a non significant effect and a significant effect at the 0.1% probability level, respectively.* | | | | | | | | | | |

**Appendix 3 |**

Mean squares for the quantification of phenolic acids and antioxidant capacity in thirteen durum wheat varieties under two farming systems (continued).

|  |  | **Diferulic acid a** | | **Diferulic acid b** | | **Diferulic acid c** | | **Triferulic acid a** | |
| --- | --- | --- | --- | --- | --- | --- | --- | --- | --- |
| **Source of variation** | **df** | **Bound** | **Total** | **Bound** | **Total** | **Bound** | **Total** | **Bound** | **Total** |
| Corrected Model | 27 | 224.22 | 224.22 | 2517.29 | 2517.29 | 19329.88 | 19329.89 | 289.70 | 289.70 |
| Constant | 1 | 16208.37 | 16208.37 | 305871.15 | 305871.15 | 2181006.98 | 2181006.98 | 53759.48 | 53759.48 |
| Replication | 2 | 1.22 | 1.22 | 14.29 | 14.29 | 20.19 | 20.31 | 13.93 | 13.93 |
| Farming system | 1 | 942.59^***^ | 942.59^***^ | 3082.23^***^ | 3082.23^***^ | 73387.18^***^ | 73387.18^***^ | 2013.51^***^ | 2013.51^***^ |
| Variety | 12 | 382.10^***^ | 382.10^***^ | 5057.58^***^ | 5057.58^***^ | 31240.52^***^ | 31240.52^***^ | 451.59^***^ | 451.59^***^ |
| Farming system * Variety | 12 | 43.64^***^ | 43.64^***^ | 347.08^***^ | 347.08^***^ | 6132.75^***^ | 6132.75^***^ | 30.12^***^ | 30.12^***^ |
| Error | 50 | 0.69 | 0.69 | 2.69 | 2.69 | 7.72 | 7.71 | 0.64 | 0.64 |
| Total | 78 |  |  |  |  |  |  |  |  |
| Corrected Total | 77 |  |  |  |  |  |  |  |  |
| **CV** |  | **32.89** | **32.89** | **46.05** | **46.05** | **44.83** | **44.83** | **33.19** | **33.19** |
| **R^2^** |  | **0.99** | **0.99** | **1.00** | **1.00** | **1.00** | **1.00** | **0.98** | **0.98** |
| **** indicates a significant effect at the 0.1% probability level.* | | | | | | | | | |

| **Appendix 4 \|**  Phenolic profile of thirteen durum wheat varieties grown under organic and conventional farming (mg/100 g) *(nd: not detected, nq: not quantified).* | | | | | | | | | | | | | | | |
| --- | --- | --- | --- | --- | --- | --- | --- | --- | --- | --- | --- | --- | --- | --- | --- |
|  | | **Caffeic acid** | | |  | **Sinapic acid** | | | **Coumaric acid** | | | **Ferulic acid** | | | |
| Varieties | Farming system | | Bound | Free | Total | Bound | Free | Total | Bound | Free | Total | Bound | Free | Total | |
| Karim | CF | | 2.89 ± 0.09 ^b^ | nd | 2.89 ± 0.09 ^b^ | 4.01 ± 0.09 ^b^ | nd | 4.01 ± 0.09 ^b^ | 4.21 ± 0.09 ^b^ | 0.96 ± 0.03 ^b^ | 5.17 ± 0.12 ^b^ | 26.69 ± 0.12 ^b^ | nd | 26.69 ± 0.12 ^b^ |  |
|  | OF | | 2.98 ± 0.02 ^a^ | nd | 2.98 ± 0.02 ^a^ | 4.29 ± 0.06 ^a^ | nd | 4.29 ± 0.06 ^a^ | 4.54 ± 0.09 ^a^ | 1.12 ± 0.10 ^a^ | 5.66 ± 0.14 ^a^ | 39.03 ± 0.15 ^a^ | nd | 39.03 ± 0.15 ^a^ | |
| Razzak | CF | | 2.72 ± 0.08 ^b^ | nd | 2.72 ± 0.08 ^b^ | 4.12 ± 0.02 ^b^ | nd | 4.12 ± 0.02 ^b^ | 3.91 ± 0.08 ^b^ | 0.87 ± 0.06 ^b^ | 4.78 ± 0.09 ^b^ | 31.90 ± 5.72 ^b^ | nd | 31.90 ± 5.72 ^b^ | |
|  | OF | | 2.89 ± 0.02 ^a^ | nd | 2.89 ± 0.02 ^a^ | 4.40 ± 0.02 ^a^ | nd | 4.40 ± 0.02 ^a^ | 4.29 ± 0.11 ^a^ | 1.20 ± 0.22 ^a^ | 5.49 ± 0.22 ^a^ | 56.11 ± 5.84 ^a^ | nd | 56.11 ± 5.84 ^a^ | |
| Khiar | CF | | 2.87 ± 0.06 ^b^ | nd | 2.87 ± 0.06 ^b^ | nq | nd | nq | 3.63 ± 0.09 ^b^ | 0.34 ± 0.07 ^b^ | 3.97 ± 0.11 ^b^ | 17.50 ± 1.01 ^a^ | nd | 17.50 ± 1.01 ^a^ | |
|  | OF | | 3.00 ± 0.10 ^a^ | nd | 3.00 ± 0.10 ^a^ | nq | nd | nq | 3.96 ± 0.06 ^a^ | 0.75 ± 0.03 ^a^ | 4.71 ± 0.08 ^a^ | 16.27 ± 0.10 ^b^ | nd | 16.27 ± 0.10 ^b^ | |
| Maali | CF | | 2.92 ± 0.07 ^b^ | nd | 2.92 ± 0.07 ^b^ | 4.09 ± 0.04 ^b^ | nd | 4.09 ± 0.04 ^b^ | 3.87 ± 0.02 ^b^ | 0.86 ± 0.08 ^a^ | 4.73 ± 0.06 ^b^ | 25.87 ± 11.55 ^a^ | nd | 25.87 ± 11.55 ^a^ | |
|  | OF | | 2.99 ± 0.01 ^a^ | nd | 2.99 ± 0.01 ^a^ | 4.32 ± 0.04 ^a^ | nd | 4.32 ± 0.04 ^a^ | 4.25 ± 0.06 ^a^ | 0.87 ± 0.06 ^a^ | 5.11 ± 0.12 ^a^ | 21.00 ± 0.10 ^b^ | nd | 21.00 ± 0.10 ^b^ | |
| Salim | CF | | nq | nd | nq | 4.20 ± 0.10 ^a^ | nd | 4.20 ± 0.10 ^a^ | 4.03 ± 0.04 ^b^ | nq | 4.03 ± 0.04 ^b^ | 44.00 ± 0.10 ^a^ | nd | 44.00 ± 0.10 ^a^ | |
|  | OF | | nq | nd | nq | 4.01 ± 0.04 ^b^ | nd | 4.01 ± 0.04 ^b^ | 4.40 ± 0.10 ^a^ | 0.93 ± 0.04 ^a^ | 5.33 ± 0.12 ^a^ | 36.29 ± 0.32 ^b^ | nd | 36.29 ± 0.32 ^b^ | |
| INRAT 100 | CF | | 2.99 ± 0.03 ^b^ | nd | 2.99 ± 0.03 ^b^ | 4.00 ± 0.10 ^b^ | nd | 4.00 ± 0.10 ^b^ | 3.81 ± 0.14 ^b^ | 0.94 ± 0.04 ^a^ | 4.75 ± 0.15 ^b^ | 33.03 ± 5.05 ^b^ | nd | 33.03 ± 5.05 ^b^ | |
|  | OF | | 3.04 ± 0.02 ^a^ | nd | 3.04 ± 0.02 ^a^ | 4.31 ± 0.03 ^a^ | nd | 4.31 ± 0.03 ^a^ | 4.17 ± 0.10 ^a^ | 0.94 ± 0.05 ^a^ | 5.11 ± 0.08 ^a^ | 39.57 ± 0.56 ^a^ | nd | 39.57 ± 0.56 ^a^ | |
| Dhahbi | CF | | 3.02 ± 0.07 ^b^ | nd | 3.02 ± 0.07 ^b^ | 4.15 ± 0.05 ^b^ | nd | 4.15 ± 0.05 ^b^ | 3.87 ± 0.06 ^b^ | 0.83 ± 0.04 ^b^ | 4.70 ± 0.03 ^b^ | 67.75 ± 4.08 ^a^ | nd | 67.75 ± 4.08 ^a^ | |
|  | OF | | 3.10 ± 0.02 ^a^ | nd | 3.10 ± 0.02 ^a^ | 4.30 ± 0.06 ^a^ | nd | 4.30 ± 0.06 ^a^ | 4.20 ± 0.11 ^a^ | 1.09 ± 0.11 ^a^ | 5.29 ± 0.10 ^a^ | 57.10 ± 0.10 ^b^ | nd | 57.10 ± 0.10 ^b^ | |
| Monastir | CF | | nq | nd | nq | 4.12 ± 0.01 ^b^ | nd | 4.12 ± 0.01 ^b^ | 3.80 ± 0.10 ^b^ | 0.89 ± 0.03 ^b^ | 4.69 ± 0.11 ^b^ | 28.13 ± 0.12 ^b^ | nd | 28.13 ± 0.12 ^b^ | |
|  | OF | | nq | nd | nq | 4.22 ± 0.04 ^a^ | nd | 4.22 ± 0.04 ^a^ | 4.14 ± 0.06 ^a^ | 0.95 ± 0.02 ^a^ | 5.08 ± 0.08 ^a^ | 30.63 ± 0.23 ^a^ | nd | 30.63 ± 0.23 ^a^ | |
| Saragolla | CF | | 2.85 ± 0.05 ^b^ | nd | 2.85 ± 0.05 ^b^ | 4.09 ± 0.02 ^b^ | nd | 4.09 ± 0.02 ^b^ | 3.83 ± 0.06 ^b^ | 0.86 ± 0.06 ^b^ | 4.69 ± 0.11 ^b^ | 23.20 ± 0.10 ^b^ | nd | 23.20 ± 0.10 ^b^ | |
|  | OF | | 2.93 ± 0.03 ^a^ | nd | 2.93 ± 0.03 ^a^ | 4.20 ± 0.04 ^a^ | nd | 4.20 ± 0.04 ^a^ | 4.24 ± 0.07 ^a^ | 0.95 ± 0.04 ^a^ | 5.19 ± 0.09 ^a^ | 46.51 ± 0.34 ^a^ | nd | 46.51 ± 0.34 ^a^ | |
| Chili | CF | | 3.02 ± 0.01 ^b^ | nd | 3.02 ± 0.01 ^b^ | 4.22 ± 0.06 ^b^ | nd | 4.22 ± 0.06 ^b^ | 4.37 ± 0.13 ^b^ | 0.87 ± 0.06 ^b^ | 5.24 ± 0.14 ^b^ | 38.59 ± 0.03 ^b^ | nd | 38.59 ± 0.03 ^b^ | |
|  | OF | | 3.10 ± 0.02 ^a^ | nd | 3.10 ± 0.02 ^a^ | 4.29 ± 0.01 ^a^ | nd | 4.29 ± 0.01 ^a^ | 4.68 ± 0.08 ^a^ | 1.09 ± 0.13 ^a^ | 5.77 ± 0.10 ^a^ | 60.46 ± 0.08 ^a^ | nd | 60.46 ± 0.08 ^a^ | |
| Biskri | CF | | 2.95 ± 0.05 ^b^ | nd | 2.95 ± 0.05 ^b^ | 4.15 ± 0.01 ^b^ | nd | 4.15 ± 0.01 ^b^ | 3.53 ± 0.06 ^b^ | 1.02 ± 0.01 ^b^ | 4.55 ± 0.08 ^b^ | 50.32 ± 1.91 ^b^ | nd | 50.32 ± 1.91 ^b^ | |
|  | OF | | 3.04 ± 0.03 ^a^ | nd | 3.04 ± 0.03 ^a^ | 4.47 ± 0.03 ^a^ | nd | 4.47 ± 0.03 ^a^ | 5.01 ± 0.14 ^a^ | 1.14 ± 0.05 ^a^ | 6.15 ± 0.19 ^a^ | 73.87 ± 1.67 ^a^ | nd | 73.87 ± 1.67 ^a^ | |
| Mahmoudi | CF | | 2.90 ± 0.06 ^b^ | nd | 2.90 ± 0.06 ^b^ | 4.27 ± 0.06 ^b^ | nd | 4.27 ± 0.06 ^b^ | 4.63 ± 0.06 ^b^ | 0.98 ± 0.04 ^b^ | 5.61 ± 0.06 ^b^ | 50.28 ± 0.80 ^b^ | nd | 50.28 ± 0.80 ^b^ | |
|  | OF | | 3.01 ± 0.06 ^a^ | nd | 3.01 ± 0.06 ^a^ | 4.38 ± 0.06 ^a^ | nd | 4.38 ± 0.06 ^a^ | 4.87 ± 0.06 ^a^ | 1.11 ± 0.04 ^a^ | 5.98 ± 0.08 ^a^ | 54.97 ± 3.62 ^a^ | nd | 54.97 ± 3.62 ^a^ | |
| Jenah Khotifa | CF | | 2.89 ± 0.03 ^b^ | nd | 2.89 ± 0.03 ^b^ | 4.16 ± 0.04 ^b^ | nd | 4.16 ± 0.04 ^b^ | 4.17 ± 0.55 ^b^ | 0.95 ± 0.04 ^a^ | 5.12 ± 0.54 ^b^ | 40.68 ± 1.56 ^b^ | nd | 40.68 ± 1.56 ^b^ | |
|  | OF | | 3.13 ± 0.02 ^a^ | nd | 3.13 ± 0.02 ^a^ | 4.24 ± 0.05 ^a^ | nd | 4.24 ± 0.05 ^a^ | 4.98 ± 0.13 ^a^ | 0.99 ± 0.01 ^a^ | 5.97 ± 0.14 ^a^ | 51.49 ± 0.45 ^a^ | nd | 51.49 ± 0.45 ^a^ | |
| **Mean** | **CF**  **OF** | | **2.91 ± 0.10 ^b^**  **3.02 ± 0.08 ^a^** | **nd**  **nd** | **2.91 ± 0.10 ^b^**  **3.02 ± 0.08 ^a^** | **4.13 ± 0.09 ^b^**  **4.29 ± 0.12 ^a^** | **nd**  **nd** | **4.13 ± 0.09 ^b^**  **4.29 ± 0.12 ^a^** | **3.97 ± 0.33 ^b^**  **4.44 ± 0.34 ^a^** | **0.86 ± 0.29 ^b^**  **1.01 ± 0.14 ^a^** | **4.77 ± 0.47 ^b^**  **5.45 ± 0.43 ^a^** | **36.76 ± 13.85 ^b^**  **44.87 ± 16.06 ^a^** | **nd**  **nd** | **36.76 ± 13.85 ^b^**  **44.87 ± 16.06 ^a^** | |
| **ANOVA** |  | |  |  |  |  |  |  |  |  |  |  |  |  | |
| Farming system |  | | ******* |  | ******* | ******* |  | ******* | ******* | ******* | ******* | ******* |  | ******* | |
| Variety |  | | ******* |  | ******* | ******* |  | ******* | ******* | ******* | ******* | ******* |  | ******* | |
| FS * Variety |  | | **NS** |  | **NS** | ******* |  | ******* | ******* | ******* | ******* | ******* |  | ******* | |
| *Values are expressed as mean ± standard deviation (n=3). Different letters within the same row indicate statistically significant differences (p ≤ 0.05, Tukey’s test)*. *CF : conventional framing ; OF : organic farming* | | | | | | | | | | | | | | | |

| **Appendix 5 \|**  Phenolic profile of thirteen durum wheat varieties grown under organic and conventional farming (mg/100 g) | | | | | | | | | | | | | |
| --- | --- | --- | --- | --- | --- | --- | --- | --- | --- | --- | --- | --- | --- |
|  | **Diferulic acid a** | | |  | **Diferulic acid b** | | | **Diferulic acid c** | | | **Triferulic acid a** | | |
| Varieties | Farming system | Bound | Free | Total | Bound | Free | Total | Bound | Free | Total | Bound | Free | Total |
| Karim | CF | 8.88 ± 1.39 ^b^ | nd | 8.88 ± 1.39 ^b^ | 40.50 ± 5.29 ^b^ | nd | 40.50 ± 5.29 ^b^ | 98.54 ± 0.81^b^ | nd | 98.54 ± 0.81 ^b^ | 12.32 ± 1.68 ^b^ | nd | 12.32 ± 1.68 ^b^ |
|  | OF | 10.08 ± 2.03 ^a^ | nd | 10.08 ± 2.03 ^a^ | 58.13 ± 1.08 ^a^ | nd | 58.13 ± 1.08 ^a^ | 175.18 ± 2.59 ^a^ | nd | 175.18 ± 2.59 ^a^ | 26.42 ± 1.67 ^a^ | nd | 26.42 ± 1.67 ^a^ |
| Razzak | CF | 9.64 ± 1.39 ^b^ | nd | 9.64 ± 1.39 ^b^ | 42.68 ± 1.32 ^b^ | nd | 42.68 ± 1.32 ^b^ | 123.60 ± 1.88 ^b^ | nd | 123.60 ± 1.88 ^b^ | 19.28 ± 1.41 ^b^ | nd | 19.28 ± 1.41 ^b^ |
|  | OF | 17.72 ± 0.48 ^a^ | nd | 17.72 ± 0.48 ^a^ | 45.92 ± 2.17 ^a^ | nd | 45.92 ± 2.17 ^a^ | 170.85 ± 7.82 ^a^ | nd | 170.85 ± 7.82 ^a^ | 36.99 ± 2.50 ^a^ | nd | 36.99 ± 2.50 ^a^ |
| Khiar | CF | nd | nd | nd | 30.00 ± 0.05 ^b^ | nd | 30.00 ± 0.05 ^b^ | 88.50 ± 0.03 ^b^ | nd | 88.50 ± 0.03 ^b^ | 10.53 ± 1.77 ^b^ | nd | 10.53 ± 1.77 ^b^ |
|  | OF | 10.87 ± 1.34 ^a^ | nd | 10.87 ± 1.34 ^a^ | 31.79 ± 1.16 ^a^ | nd | 31.79 ± 1.16 ^a^ | 92.39 ± 5.73 ^a^ | nd | 92.39 ± 5.73 ^a^ | 16.99 ± 0.64 ^a^ | nd | 16.99 ± 0.64 ^a^ |
| Maali | CF | 13.35 ± 0.04 ^b^ | nd | 13.35 ± 0.04 ^b^ | 77.95 ± 1.00 ^b^ | nd | 77.95 ± 1.00 ^b^ | 221.25 ± 0.10 ^b^ | nd | 221.25 ± 0.10 ^b^ | 21.65 ± 1.04 ^b^ | nd | 21.65 ± 1.04 ^b^ |
|  | OF | 14.55 ± 0.22 ^a^ | nd | 14.55 ± 0.22 ^a^ | 85.69 ± 1.37 ^a^ | nd | 85.69 ± 1.37 ^a^ | 224.99 ± 2.65 ^a^ | nd | 224.99 ± 2.65 ^a^ | 35.86 ± 0.87 ^a^ | nd | 35.86 ± 0.87 ^a^ |
| Salim | CF | nq | nd | nq | 29.18 ± 0.96 ^a^ | nd | 29.18 ± 0.96 ^a^ | 59.50 ± 0.12 ^b^ | nd | 59.50 ± 0.12 ^b^ | 8.69 ± 1.07 ^b^ | nd | 8.69 ± 1.07 ^b^ |
|  | OF | nq | nd | nq | 20.36 ± 2.13 ^b^ | nd | 20.36 ± 2.13 ^b^ | 80.18 ± 0.91 ^a^ | nd | 80.18 ± 0.91 ^a^ | 10.73 ± 0.93 ^a^ | nd | 10.73 ± 0.93 ^a^ |
| INRAT 100 | CF | nq | nd | nq | 64.83 ± 2.04 ^b^ | nd | 64.83 ± 2.04 ^b^ | 125.70 ± 2.36 ^b^ | nd | 125.70 ± 2.36 ^b^ | 26.74 ± 0.30 ^b^ | nd | 26.74 ± 0.30 ^b^ |
|  | OF | 16.33 ± 0.39 ^a^ | nd | 16.33 ± 0.39 ^a^ | 65.26 ± 1.82 ^a^ | nd | 65.26 ± 1.82 ^a^ | 200.60 ± 6.87 ^a^ | nd | 200.60 ± 6.87 ^a^ | 38.74 ± 0.73 ^a^ | nd | 38.74 ± 0.73 ^a^ |
| Dhahbi | CF | 24.35 ± 0.94 ^b^ | nd | 24.35 ± 0.94 ^b^ | 58.16 ± 0.96 ^a^ | nd | 58.16 ± 0.96 ^a^ | 267.91 ± 1.82 ^b^ | nd | 267.91 ± 1.82 ^b^ | 30.74 ± 0.49 ^b^ | nd | 30.74 ± 0.49 ^b^ |
|  | OF | 26.76 ± 0.57 ^a^ | nd | 26.76 ± 0.57 ^a^ | 58.55 ± 1.33 ^a^ | nd | 58.55 ± 1.33 ^a^ | 284.47 ± 3.05 ^a^ | nd | 284.47 ± 3.05 ^a^ | 36.86 ± 0.59 ^a^ | nd | 36.86 ± 0.59 ^a^ |
| Monastir | CF | nq | nd | nq | 30.50 ± 0.06 ^b^ | nd | 30.50 ± 0.06 ^b^ | 77.90 ± 1.65 ^b^ | nd | 77.90 ± 1.65 ^b^ | 12.55 ± 0.48 ^b^ | nd | 12.55 ± 0.48 ^b^ |
|  | OF | 13.34 ± 0.78 ^a^ | nd | 13.34 ± 0.78 ^a^ | 39.07 ± 0.23 ^a^ | nd | 39.07 ± 0.23 ^a^ | 82.32 ± 1.12 ^a^ | nd | 82.32 ± 1.12 ^a^ | 20.48 ± 0.04 ^a^ | nd | 20.48 ± 0.04 ^a^ |
| Saragolla | CF | 21.70 ± 1.12 ^b^ | nd | 21.70 ± 1.12 ^b^ | 26.56 ± 0.31 ^b^ | nd | 26.56 ± 0.31 ^b^ | 94.36 ± 2.05 ^b^ | nd | 94.36 ± 2.05 ^b^ | 23.41 ± 0.44 ^b^ | nd | 23.41 ± 0.44 ^b^ |
|  | OF | 23.48 ± 0.42 ^a^ | nd | 23.48 ± 0.42 ^a^ | 44.56 ± 1.78 ^a^ | nd | 44.56 ± 1.78 ^a^ | 98.45 ± 1.46 ^a^ | nd | 98.45 ± 1.46 ^a^ | 28.59 ± 0.42 ^a^ | nd | 28.59 ± 0.42 ^a^ |
| Chili | CF | 19.14 ± 0.65 ^b^ | nd | 19.14 ± 0.65 ^b^ | 73.26 ± 0.64 ^b^ | nd | 73.26 ± 0.64 ^b^ | 142.72 ± 2.07 ^b^ | nd | 142.72 ± 2.07 ^b^ | 26.69 ± 0.54 ^b^ | nd | 26.69 ± 0.54 ^b^ |
|  | OF | 25.50 ± 0.75 ^a^ | nd | 25.50 ± 0.75 ^a^ | 98.12 ± 0.12 ^a^ | nd | 98.12 ± 0.12 ^a^ | 334.53 ± 3.12 ^a^ | nd | 334.53 ± 3.12 ^a^ | 41.39 ± 0.54 ^a^ | nd | 41.39 ± 0.54 ^a^ |
| Biskri | CF | 15.73 ± 1.01 ^b^ | nd | 15.73 ± 1.01 ^b^ | 74.85 ± 0.04 ^b^ | nd | 74.85 ± 0.04 ^b^ | 188.79 ± 0.53 ^b^ | nd | 188.79 ± 0.53 ^b^ | 29.04 ± 0.16 ^b^ | nd | 29.04 ± 0.16 ^b^ |
|  | OF | 24.57 ± 1.46 ^a^ | nd | 24.57 ± 1.46 ^a^ | 125.40 ± 3.36 ^a^ | nd | 125.40 ± 3.36 ^a^ | 348.97 ± 1.17 ^a^ | nd | 348.97 ± 1.17 ^a^ | 39.05 ± 0.82 ^a^ | nd | 39.05 ± 0.82 ^a^ |
| Mahmoudi | CF | 14.07 ± 0.26 ^b^ | nd | 14.07 ± 0.26 ^b^ | 82.44 ± 2.45 ^b^ | nd | 82.44 ± 2.45 ^b^ | 132.47 ± 0.38 ^b^ | nd | 132.47 ± 0.38 ^b^ | 22.49 ± 1.55 ^b^ | nd | 22.49 ± 1.55 ^b^ |
|  | OF | 20.95 ± 0.64 ^a^ | nd | 20.95 ± 0.64 ^a^ | 97.77 ± 1.88 ^a^ | nd | 97.77 ± 1.88 ^a^ | 194.77 ± 1.80 ^a^ | nd | 194.77 ± 1.80 ^a^ | 34.19 ± 1.02 ^a^ | nd | 34.19 ± 1.02 ^a^ |
| Jenah Khotifa | CF | 15.34 ± 0.47 ^b^ | nd | 15.34 ± 0.47 ^b^ | 101.45 ± 0.88 ^b^ | nd | 101.45 ± 0.88 ^b^ | 153.83 ± 1.99 ^b^ | nd | 153.83 ± 1.99 ^b^ | 31.11 ± 0.50 ^b^ | nd | 31.11 ± 0.50 ^b^ |
|  | OF | 28.43 ± 0.50 ^a^ | nd | 28.43 ± 0.50 ^a^ | 125.17 ± 0.81^a^ | nd | 125.17 ± 0.81 ^a^ | 284.88 ± 1.33 ^a^ | nd | 284.88 ± 1.33 ^a^ | 41.05 ± 1.40 ^a^ | nd | 41.05 ± 1.40 ^a^ |
| **Mean** | **CF**  **OF** | **15.80 ± 8.48 ^b^**  **19.38 ± 7.97 ^a^** | **nd**  **nd** | **15.80 ± 8.48 ^b^**  **19.38 ± 7.97 ^a^** | **56.34 ± 24.07 ^b^**  **68.91 ± 33.64 ^a^** | **nd**  **nd** | **56.34 ± 24.07 ^b^**  **68.91 ± 33.64 ^a^** | **136.54 ± 58.08 ^b^**  **197.89 ± 91.87 ^a^** | **nd**  **nd** | **136.54 ± 58.08 ^b^**  **197.89 ± 91.87 ^a^** | **21.17 ± 7.72 ^b^**  **31.33 ± 9.70 ^a^** | **nd**  **nd** | **21.17 ± 7.72 ^b^**  **31.33 ± 9.70 ^a^** |
| **ANOVA** |  |  |  |  |  |  |  |  |  |  |  |  |  |
| Farming system |  | ******* |  | ******* | ******* |  | ******* | ******* |  | ******* | ******* |  | ******* |
| Variety |  | ******* |  | ******* | ******* |  | ******* | ******* |  | ******* | ******* |  | ******* |
| FS * Variety |  | ******* |  | ******* | ******* |  | ******* | ******* |  | ******* | ******* |  | ******* |
| *Values are expressed as mean ± standard deviation (n=3). Different letters within the same row indicate statistically significant differences (p ≤ 0.05, Tukey’s test). CF: conventional farming; OF: organic farming* | | | | | | | | | | | | | |

**Appendix 6 |**

Mean squares for the quantification of phenolic acids and antioxidant capacity in thirteen durum wheat varieties under two farming systems.

|  |  | **Total diferulic acid** | | **Total Ferulic acid** | | **FRAP** | | |
| --- | --- | --- | --- | --- | --- | --- | --- | --- |
| **Source of variation** | **df** | **Bound** | **Total** | **Bound** | **Total** | **Free** | **Bound** | **Total** |
| Corrected Model | 27 | 36831.02 | 36831.02 | 50671.20 | 50671.20 | 0.00 | 0.44 | 0.48 |
| Constant | 1 | 4653475.43 | 4653475.43 | 7559932.99 | 7559932.99 | 2.07 | 542.31 | 611.30 |
| Replication | 2 | 87.53 | 87.53 | 256.45 | 256.45 | 0.00 | 0.00 | 0.00 |
| Farming system | 1 | 127534.82^***^ | 127534.82^***^ | 191664.39^***^ | 191664.39^***^ | 0.01^***^ | 0.63^***^ | 0.78^***^ |
| Variety | 12 | 63300.96^***^ | 63300.96^***^ | 86511.83^***^ | 86511.83^***^ | 0.00^***^ | 0.83^***^ | 0.89^***^ |
| Farming system * Variety | 12 | 8926.35^***^ | 8926.35^***^ | 11483.60^***^ | 11483.60^***^ | 0.00^***^ | 0.11^***^ | 0.11^***^ |
| Error | 50 | 15.17 | 15.17 | 30.90 | 30.90 | 0.00 | 0.01 | 0.01 |
| Total | 78 |  |  |  |  |  |  |  |
| Corrected Total | 77 |  |  |  |  |  |  |  |
| **CV** |  | **45.78** | **45.78** | **41.67** | **41.67** | **22.13** | **14.77** | **14.29** |
| **R^2^** |  | **1.00** | **1.00** | **1.00** | **1.00** | **0.93** | **0.97** | **0.97** |
| **** indicates a significant effect at the 0.1% probability level.* | | | | | | | | |

**Appendix 7 |**

Phytochemical Profile of durum wheat genotypes.

|  | **Total diferulic acid** | | | |  | **Total ferulic acid** | | |
| --- | --- | --- | --- | --- | --- | --- | --- | --- |
| Varieties | | Farming system | Bound | Free | Total | Bound | Free | Total |
| Karim | | CF | 147.92 ± 6.36 ^b^ | nd | 147.92 ± 6.36 ^b^ | 186.93 ± 8.14 ^b^ | nd | 186.93 ± 8.14 ^b^ |
|  | | OF | 243.39 ± 0.46 ^a^ | nd | 243.39 ± 0.46 ^a^ | 308.85 ± 1.47 ^a^ | nd | 308.85 ± 1.47 ^a^ |
| Razzak | | CF | 175.92 ± 3.73 ^b^ | nd | 175.92 ± 3.73 ^b^ | 227.09 ± 10.52 ^b^ | nd | 227.09 ± 10.52 ^b^ |
|  | | OF | 234.49 ± 9.68 ^a^ | nd | 234.49 ± 9.68 ^a^ | 327.59 ± 16.62 ^a^ | nd | 327.59 ± 16.62 ^a^ |
| Khiar | | CF | 118.50 ± 0.02 ^b^ | nd | 118.50 ± 0.02 ^b^ | 146.52 ± 2.76 ^b^ | nd | 146.52 ± 2.76 ^b^ |
|  | | OF | 135.05 ± 8.15 ^a^ | nd | 135.05 ± 8.15 ^a^ | 168.31 ± 8.00 ^a^ | nd | 168.31 ± 8.00 ^a^ |
| Maali | | CF | 312.55 ± 0.98 ^b^ | nd | 312.55 ± 0.98 ^b^ | 360.07 ± 9.99 ^b^ | nd | 360.07 ± 9.99 ^b^ |
|  | | OF | 325.24 ± 3.93 ^a^ | nd | 325.24 ± 3.93 ^a^ | 382.10 ± 3.45 ^a^ | nd | 382.10 ± 3.45 ^a^ |
| Salim | | CF | 88.68 ± 0.90 ^b^ | nd | 88.68 ± 0.90 ^b^ | 141.37 ± 1.49 ^b^ | nd | 141.37 ± 1.49 ^b^ |
|  | | OF | 100.54 ± 3.01 ^a^ | nd | 100.54 ± 3.01 ^a^ | 147.56 ± 4.25 ^a^ | nd | 147.56 ± 4.25 ^a^ |
| INRAT 100 | | CF | 190.53 ± 4.34 ^b^ | nd | 190.53 ± 4.34 ^b^ | 250.30 ± 8.99 ^b^ | nd | 250.30 ± 8.99 ^b^ |
|  | | OF | 282.19 ± 8.81 ^a^ | nd | 282.19 ± 8.81 ^a^ | 360.50 ± 8.61 ^a^ | nd | 360.50 ± 8.61 ^a^ |
| Dhahbi | | CF | 350.42 ± 1.85 ^b^ | nd | 350.42 ± 1.85 ^b^ | 448.91 ± 6.11 ^b^ | nd | 448.91 ± 6.11 ^b^ |
|  | | OF | 369.78 ± 3.40 ^a^ | nd | 369.78 ± 3.40 ^a^ | 463.74 ± 3.99 ^a^ | nd | 463.74 ± 3.99 ^a^ |
| Monastir | | CF | 108.40 ± 1.68 ^b^ | nd | 108.40 ± 1.68 ^b^ | 149.08 ± 1.78 ^b^ | nd | 149.08 ± 1.78 ^b^ |
|  | | OF | 134.73 ± 1.19 ^a^ | nd | 134.73 ± 1.19 ^a^ | 185.84 ± 1.24 ^a^ | nd | 185.84 ± 1.24 ^a^ |
| Saragolla | | CF | 142.63 ± 2.73 ^b^ | nd | 142.63 ± 2.73 ^b^ | 189.23 ± 2.61 ^b^ | nd | 189.23 ± 2.61 ^b^ |
|  | | OF | 166.49 ± 3.28 ^a^ | nd | 166.49 ± 3.28 ^a^ | 241.59 ± 2.53 ^a^ | nd | 241.59 ± 2.53 ^a^ |
| Chili | | CF | 235.12 ± 2.14 ^b^ | nd | 235.12 ± 2.14 ^b^ | 300.39 ± 2.10 ^b^ | nd | 300.39 ± 2.10 ^b^ |
|  | | OF | 458.15 ± 3.87 ^a^ | nd | 458.15 ± 3.87 ^a^ | 560.00 ± 3.72 ^a^ | nd | 560.00 ± 3.72 ^a^ |
| Biskri | | CF | 279.37 ± 1.40 ^b^ | nd | 279.37 ± 1.40 ^b^ | 358.73 ± 0.36 ^b^ | nd | 358.73 ± 0.36 ^b^ |
|  | | OF | 498.94 ± 5.87 ^a^ | nd | 498.94 ± 5.87 ^a^ | 611.85 ± 5.51 ^a^ | nd | 611.85 ± 5.51 ^a^ |
| Mahmoudi | | CF | 228.97 ± 3.04 ^b^ | nd | 228.97 ± 3.04 ^b^ | 301.74 ± 1.78 ^b^ | nd | 301.74 ± 1.78 ^b^ |
|  | | OF | 313.49 ± 4.23 ^a^ | nd | 313.49 ± 4.23 ^a^ | 402.66 ± 8.82 ^a^ | nd | 402.66 ± 8.82 ^a^ |
| Jenah Khotifa | | CF | 270.62 ± 2.47 ^b^ | nd | 270.62 ± 2.47 ^b^ | 342.41 ± 3.77 ^b^ | nd | 342.41 ± 3.77 ^b^ |
|  | | OF | 438.48 ± 1.70 ^a^ | nd | 438.48 ± 1.70 ^a^ | 531.02 ± 0.64 ^a^ | nd | 531.02 ± 0.64 ^a^ |
| **Mean** | | **CF**  **OF** | **203.82 ± 80.77 ^b^**  **284.69 ± 127.71 ^a^** | **nd**  **nd** | **203.82 ± 80.77 ^b^**  **284.69 ± 127.71 ^a^** | **261.75 ± 95.96 ^b^**  **360.89 ± 147.62 ^a^** | **nd**  **nd** | **261.75 ± 95.96 ^b^**  **360.89 ± 147.62 ^a^** |
| **ANOVA** | |  |  |  |  |  |  |  |
| Farming system | |  | ******* |  | ******* | ******* |  | ******* |
| Variety | |  | ******* |  | ******* | ******* |  | ******* |
| FS * Variety | |  | ******* |  | ******* | ******* |  | ******* |
| *Values are expressed as mean ± standard deviation (n=3). Different letters within the same row indicate statistically significant differences (p ≤ 0.05, Tukey’s test). CF: conventional farming; OF: organic farming* | | | | | | | | |
